# Inhibition of Ferroptosis Delays Aging and Extends Healthspan Across Multiple Species

**DOI:** 10.1002/advs.202416559

**Published:** 2025-03-31

**Authors:** Hai‐Jun Fu, Xing‐Yue Zhou, Da‐Lian Qin, Qan Qiao, Qiao‐Zhi Wang, Shi‐Ying Li, Yun‐Fei Zhu, Ya‐Ping Li, Jiang‐Min Zhou, Hui Cai, Fei‐Hong Huang, Lu Yu, Long Wang, An‐Guo Wu, Jian‐Ming Wu, Xiao‐Gang Zhou

**Affiliations:** ^1^ Sichuan Key Medical Laboratory of New Drug Discovery and Drugability Evaluation Luzhou Key Laboratory of Activity Screening and Druggability Evaluation for Chinese Materia Medica Key Laboratory of Medical Electrophysiology of Ministry of Education School of Pharmacy Southwest Medical University Luzhou Sichuan 646000 China; ^2^ Central Nervous System Drug Key Laboratory of Sichuan Province Luzhou Sichuan 646000 China; ^3^ School of Basic Medical Sciences Southwest Medical University Luzhou Sichuan 646000 China

**Keywords:** cellular senescence, ferroptosis, ferrostatin‐1, glutathione peroxidase 4, healthspan

## Abstract

Ferroptosis, a form of iron‐dependent cell death, plays a pivotal role in age‐related diseases; yet, its impact on cellular senescence and healthspan in mammals remains largely unexplored. This study identifies ferroptosis as a key regulator of cellular senescence, showing that its inhibition can significantly delay aging and extend healthspan across multiple species. During cellular senescence, ferroptosis is progressively exacerbated, marked by increased lipid peroxidation, oxidative stress, and diminished glutathione peroxidase 4 (GPX4) levels. Ferroptosis inducers such as Erastin and RSL3 accelerate senescence; while, inhibitors such as liproxstatin‐1 (Lip‐1) and ferrostatin‐1 (Fer‐1) effectively mitigate both chemically and replicatively induced senescence. In vivo, Fer‐1 extends lifespan and healthspan in *Caenorhabditis elegans*, enhances motor function, preserves tissue integrity, and mitigates cognitive decline in both prematurely and naturally aged mice. These effects are attributed to Fer‐1's upregulation of GPX4 and inhibition of ferroptosis. Notably, long‐term Fer‐1 treatment (over 6 months) does not adversely affect body weight or induce aging‐related tissue damage but rejuvenates hematological parameters. These findings establish ferroptosis as a critical player in aging dynamics and highlight its inhibition as a promising strategy to extend healthspan and lifespan, providing valuable insights for translational approaches to combat aging and age‐related decline.

## Graphical abstract

This study identifies ferroptosis as a key driver of cellular senescence, with its inhibition delaying aging and extending healthspan across species. During senescence, ferroptosis worsens, increasing oxidative stress and lipid peroxidation, which accelerate aging. Fer‐1 counteracts these effects, extending lifespan, preserving tissue integrity, and improving motor and cognitive function in *Caenorhabditis elegans* and aging mice without adverse effects.

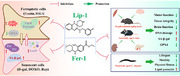

## 1 Introduction

Aging is a multifaceted and irreversible process characterized by a gradual decline in physiological functions and an increased susceptibility to various diseases.^[^ ^1^ ^]^ Growing evidence links aging to significant changes in key biological processes, including elevated senescence‐associated β‐galactosidase (SA‐β‐gal) activity, reduced antioxidant capacity, and activation of the senescence‐associated secretory phenotype (SASP).^[^ ^2^ ^]^ These biomarkers not only drive aging but also increase the risk of age‐related conditions such as kidney disease,^[^ ^3^ ^]^ osteoporosis,^[^ ^4^ ^]^ cardiovascular disease,^[^ ^5^ ^]^ metabolic syndrome,^[^ ^6^ ^]^ and neurodegenerative disorders,^[^ ^7^ ^]^ all of which contribute to higher mortality rates in older adults. Given the rapid global increase in the aging population, addressing age‐related health challenges has become increasingly urgent.^[^ ^8^ ^]^ Understanding the underlying mechanisms of aging and their connection to age‐related diseases is crucial for developing effective prevention strategies and therapeutic interventions, ultimately extending healthspan and improving quality of life.

Ferroptosis is a distinct form of iron‐dependent programmed cell death, driven by intracellular iron accumulation and lipid peroxidation.^[^ ^9^ ^]^ Unlike apoptosis, necrosis, and autophagy, ferroptosis exhibits unique biochemical and morphological features, including cell swelling, dense mitochondrial electron deposits, rupture of the outer mitochondrial membrane, and mitochondrial shrinkage or loss.^[^ ^10^ ^]^ The molecular mechanisms of ferroptosis involve the glutamate/cystine antiporter, the antioxidant system (particularly glutathione peroxidase 4, GPX4), unsaturated fatty acid metabolism, iron homeostasis, and the ferroptosis suppressor protein 1 (FSP1)‐ubiquinone system.^[^ ^11^ ^]^ Recent research underscores the critical role of ferroptosis in aging. With advancing age, iron accumulation and mitochondrial dysfunction create a feedback loop that exacerbates cellular and tissue damage, accelerating the aging process.^[^ ^12^ ^]^ While iron is essential for physiological functions such as red blood cell production, enzyme activity, and cellular energy generation, its homeostasis becomes dysregulated with age.^[^ ^13^ ^]^ This disruption leads to iron overload in tissues, triggering ferroptosis, which is strongly linked to age‐related diseases, including neurodegenerative disorders such as Alzheimer's and Parkinson's,^[^ ^14^ ^]^ as well as bone resorption.^[^ ^15^ ^]^ In addition, the accumulation of dysfunctional mitochondria increases oxidative stress, elevating reactive oxygen species (ROS) levels. Over time, cells become less capable of managing oxidative stress, particularly lipid peroxides, which act as key ferroptosis triggers.^[^ ^16^ ^]^ When lipid peroxides reach toxic levels, they induce cell death, further contributing to aging.^[^ ^17^ ^]^ Consequently, the progressive accumulation of iron and lipid peroxides highlights ferroptosis as a pivotal factor in aging.

Recent studies suggest that inhibiting ferroptosis, either by preventing lipid peroxidation or regulating iron accumulation, extends lifespan and healthspan in *Caenorhabditis elegans* and delays aging in vascular and liver tissues of mice.^[^ ^18^ ^]^ However, the precise role of ferroptosis in cellular senescence and systemic aging in mammals remains unclear, with limited direct evidence linking its inhibition to improved healthspan. This study aims to bridge these knowledge gaps by exploring the complex relationship between ferroptosis and aging across multiple models, providing novel insights and potential strategies for developing effective anti‐aging therapies.

## 2 Results

### 2.1 Cellular Senescence is Accompanied by Ferroptosis

**Figure 1:**
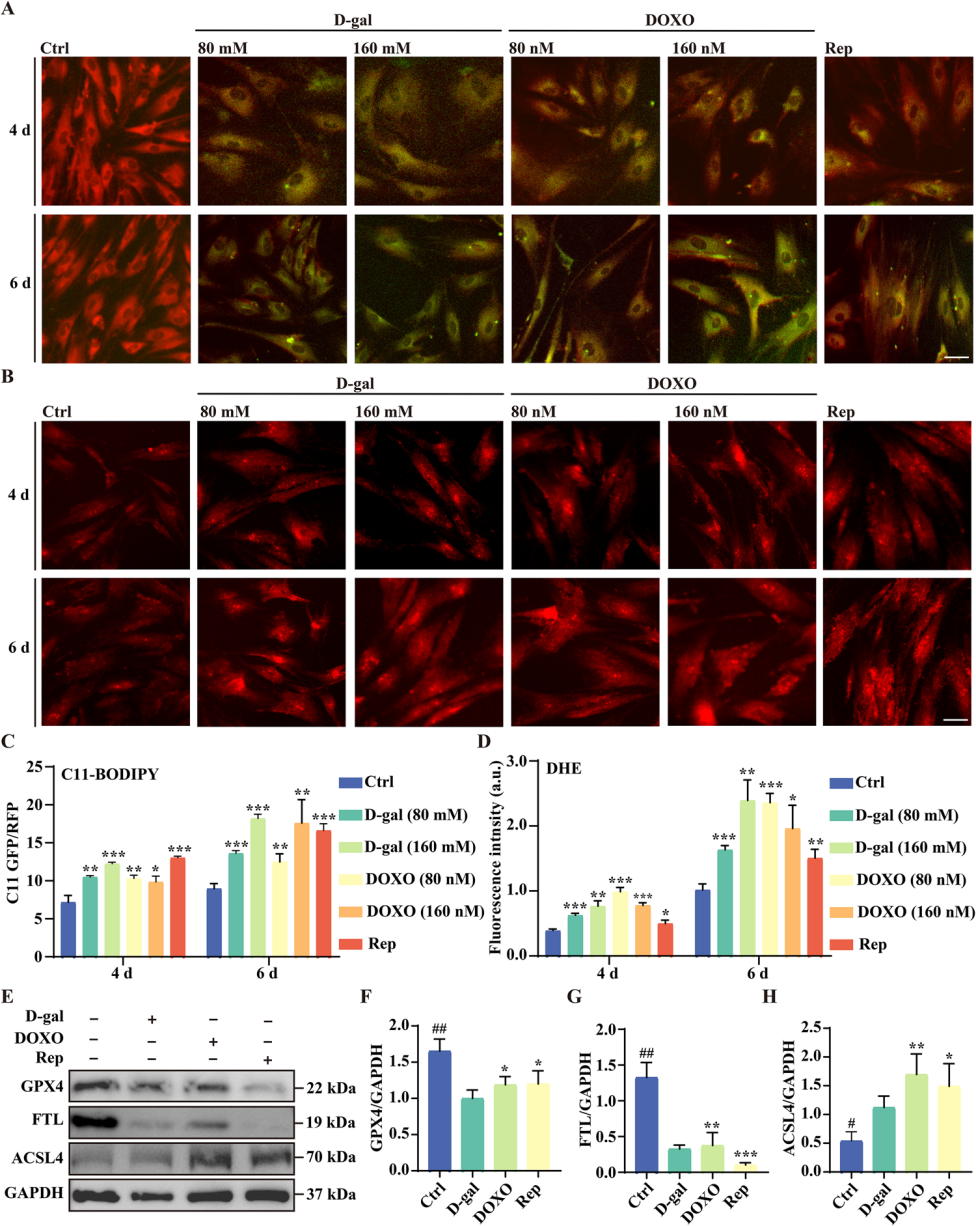
Cellular senescence is associated with ferroptosis. A,B) BODIPY 581/591 C11 and DHE staining of HFF cells treated with D‐gal (80 and 160 mm) and DOXO (80 and 160 nm), along with replicative senescent HFF cells (30 passages). Scale bar: 50 µm. C,D) Quantification of fluorescence intensity in (A,B), presented as mean ± SD from three independent experiments. Statistical significance: *p < 0.05, **p < 0.01, and ***p < 0.001. E,F) Western blot analysis of GPX4, FTL, and ACSL4 protein expression in HFF cells under different aging conditions. Bar graphs represent quantified protein levels, shown as mean ± SD from three independent experiments. Statistical significance: *p < 0.05, **p < 0.01, and **p < 0.001.

To investigate whether ferroptosis occurs during cellular senescence, we established three senescence models using primary HFF cells. Two models were induced by acute senescence inducers, D‐gal and DOXO, which are well‐established for studying cellular senescence. After confirming the cytotoxic effects of these inducers on HFF cells (Figure S1A,B, Supporting information), we treated the cells with 80 and 160 mm D‐gal, and with 80 and 160 nm DOXO. Both treatments resulted in a significant increase in SA‐β‐gal‐positive cells (Figure S1C,D, Supporting information), confirming successful senescence induction. The third model involved inducing replicative senescence by passaging HFF cells for 30 passages (Figure S1C,D, Supporting information). To assess ferroptosis during cellular senescence, we used C11‐BODIPY, a lipid peroxidation probe sensitive to ferroptosis. A time‐dependent increase in C11‐BODIPY fluorescence intensity was observed across all three senescence models, with stronger signals at day 6 compared to day 4 (**Figure** **1**A,C). In addition, C11‐BODIPY fluorescence intensity in D‐gal‐ and DOXO‐treated HFF cells was concentration‐dependent (Figure 1A,C). To further confirm ferroptosis, we measured ROS levels using DHE staining. ROS levels increased in all three models (Figure 1B,D), paralleling the rise in lipid peroxidation. Further, the expression of ferroptosis‐related proteins, including GPX4 and FTL, was downregulated in all three senescence models; while, ACSL4 expression was upregulated (Figure 1E–H). Collectively, these findings confirm that ferroptosis is associated with cellular senescence.

### 2.2 Ferroptosis Inducers Erastin and RSL3 Accelerate Cellular Senescence

**Figure 2:**
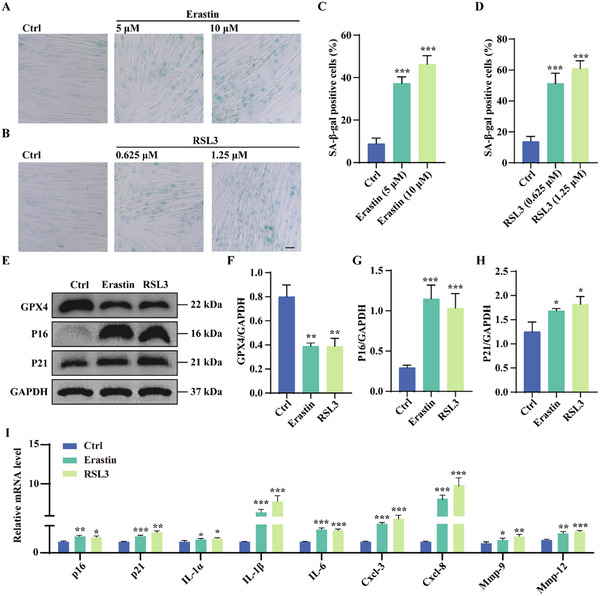
Ferroptosis inducers Erastin and RSL3 accelerate cellular senescence. A,B) SA‐β‐gal staining in HFF cells treated with Erastin (5 and10 µm) and RSL3 (0.625 and 1.25 µm) to induce senescence. Representative images show control and senescent HFF cells. Scale bar: 100 µm. C,D) Quantification of SA‐β‐gal‐positive cells in (A,B). Data are presented as mean ± SD from three independent experiments. Statistical significance: **p < 0.01 and ***p < 0.001. E–H) Western blot analysis of GPX4, P16, and P21 protein levels in HFF cells treated with Erastin and RSL3. Bar graphs show quantification of GPX4, P16, and P21 protein expression. Data are expressed as mean ± SD from three independent experiments. Statistical significance: *p < 0.05, **p < 0.01, and ***p < 0.001. I) Quantification of p16, p21, and SASP gene mRNA levels in HFF cells treated with Erastin and RSL3 compared to controls. Data are shown as mean ± SD from three independent experiments. Statistical significance: *p < 0.05, **p < 0.01, and ***p < 0.001.

To further elucidate the relationship between ferroptosis and cellular senescence, we treated HFF cells with the ferroptosis inducers Erastin (5–10 µm) and RSL3 (0.625–1.25 µm) at non‐toxic concentrations for 5 days (Figure S2, Supporting information). Senescent cell numbers were assessed using SA‐β‐gal staining. By day 5, both the Erastin and RSL3‐treated groups exhibited a dose‐dependent increase in senescent cells compared to the control group (**Figure** **2**A–D). We next examined the expression of key senescence markers, P16 and P21, via Western blotting. Erastin and RSL3 treatment significantly downregulated GPX4 expression (Figure 2E,F), confirming ferroptosis induction. Concurrently, the expression of senescence markers P16 and P21 was markedly upregulated in treated groups (Figure 2E,G,H). In addition, qRT‐PCR analysis revealed elevated mRNA levels of *p16*, *p21*, and SASP factors, including *IL‐1α*, *IL‐1β*, *IL‐6*, *Cxcl‐3*, *Cxcl‐8*, *Mmp‐9*, and *Mmp‐12* following Erastin and RSL3 treatment (Figure 2I). Together, these findings highlight the critical role of ferroptosis in driving cellular senescence, confirming that ferroptosis inducers accelerate the onset of senescence.

### 2.3 Ferroptosis Inhibitors Fer‐1 and Lip‐1 Mitigate Cellular Senescence Induced by Multiple Stressors

**Figure 3:**
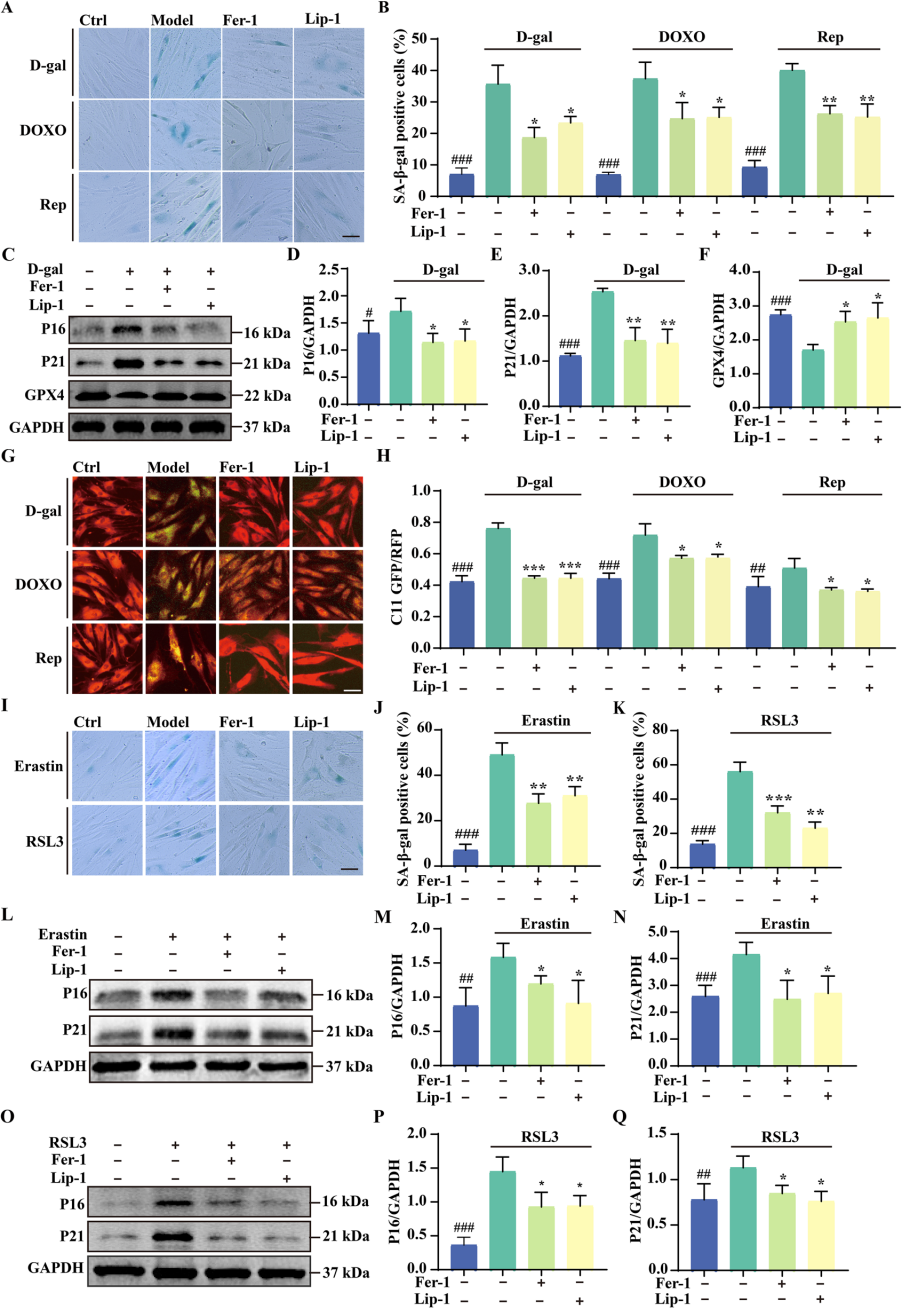
Ferroptosis inhibitors Fer‐1 and Lip‐1 mitigate senescence induced by various triggers. A,B) SA‐β‐gal staining in HFF cells under three aging conditions, with or without Fer‐1 (1 µm) and Lip‐1 (1 µm) treatment. The bar graph shows the percentage of SA‐β‐gal‐positive cells. Data are expressed as mean ± SD from three independent experiments. Statistical significance: *p < 0.05, **p < 0.01, and ***p < 0.001. # indicates a significant difference between the control and model groups. Scale bar: 25 µm. C) Western blot analysis of GPX4, P16, and P21 protein levels in D‐gal‐induced senescent HFF cells treated with Fer‐1 and Lip‐1. D–F) Bar graphs quantify GPX4, P16, and P21 expression. Data are presented as mean ± SD from three independent experiments. Statistical significance: *p < 0.05, **p < 0.01, and ***p < 0.001. # indicates a significant difference between the control and model groups. G,H) Representative images of BODIPY 581/591 C11 staining in HFF cells under D‐gal‐ and DOXO‐induced senescence or replicative senescence, with or without Fer‐1 and Lip‐1. The bar graph quantifies the C11 GFP/RFP ratio. Data are expressed as mean ± SD from three independent experiments. Statistical significance: *p < 0.05, **p < 0.01, and ***p < 0.001. # indicates a significant difference between the control and model groups. Scale bar: 25 µm. I–K) SA‐β‐gal staining in HFF cells treated with Erastin and RSL3, with or without Fer‐1 and Lip‐1. The bar graph quantifies the percentage of SA‐β‐gal‐positive cells. Data are presented as mean ± SD from three independent experiments. Statistical significance: **p < 0.01 and ***p < 0.001. # indicates a significant difference between the control and model groups. Scale bar: 25 µm. L–N) Western blot analysis of P16 and P21 protein levels in Erastin‐induced senescent HFF cells treated with Fer‐1 and Lip‐1. Data are shown as mean ± SD from three independent experiments, with *p < 0.05, **p < 0.01, and ***p < 0.001. # indicates a significant difference between the control and model groups. O–Q) Western blot analysis of P16 and P21 protein levels in RSL3‐induced senescent HFF cells treated with Fer‐1 and Lip‐1. Data are presented as mean ± SD from three independent experiments, with *p < 0.05, **p < 0.01, and ***p < 0.001. # indicates a significant difference between the control and model groups.

To determine whether ferroptosis inhibition can delay cellular senescence, we treated three senescence models with the ferroptosis inhibitors Fer‐1 and Lip‐1, assessing their effects using SA‐β‐gal staining. Both inhibitors significantly reduced the number of senescent cells in D‐gal, DOXO, and replicative stress‐induced models, as indicated by a decrease in SA‐β‐gal‐positive cells compared to untreated controls (**Figure** **3**A,B). Western blot analysis further confirmed that Fer‐1 and Lip‐1 effectively reduced the elevated levels of senescence markers P16 and P21 induced by D‐gal treatment (Figure 3C–E), suggesting that these inhibitors can delay cellular senescence. Given the established link between ferroptosis and senescence, we next examined whether Fer‐1 and Lip‐1 could mitigate ferroptosis in these models. Treatment with both inhibitors restored GPX4 expression, which had been downregulated in D‐gal‐treated HFF cells (Figure 3C,F). In addition, both inhibitors significantly reduced lipid peroxidation, as measured by C11‐BODIPY fluorescence intensity, across all three senescence models (Figure 3G,H).

We further investigated whether Fer‐1 and Lip‐1 could counteract senescence induced by the ferroptosis inducers Erastin and RSL3. SA‐β‐gal staining revealed a marked reduction in the proportion of senescent cells in groups treated with Fer‐1 and Lip‐1 following ferroptosis inducer exposure (Figure 3I–K). Western blot analysis showed that these inhibitors significantly decreased the expression of P16 and P21, which had been upregulated by Erastin and RSL3 (Figure 3L–Q). Together, these findings demonstrate that Fer‐1 and Lip‐1 effectively delay cellular senescence induced by various stressors, including chemical inducers, replicative stress, and ferroptosis inducers.

### 2.4 Fer‐1 Enhances Healthspan and Prevents Ferroptosis in *C. elegans*

**Figure 4:**
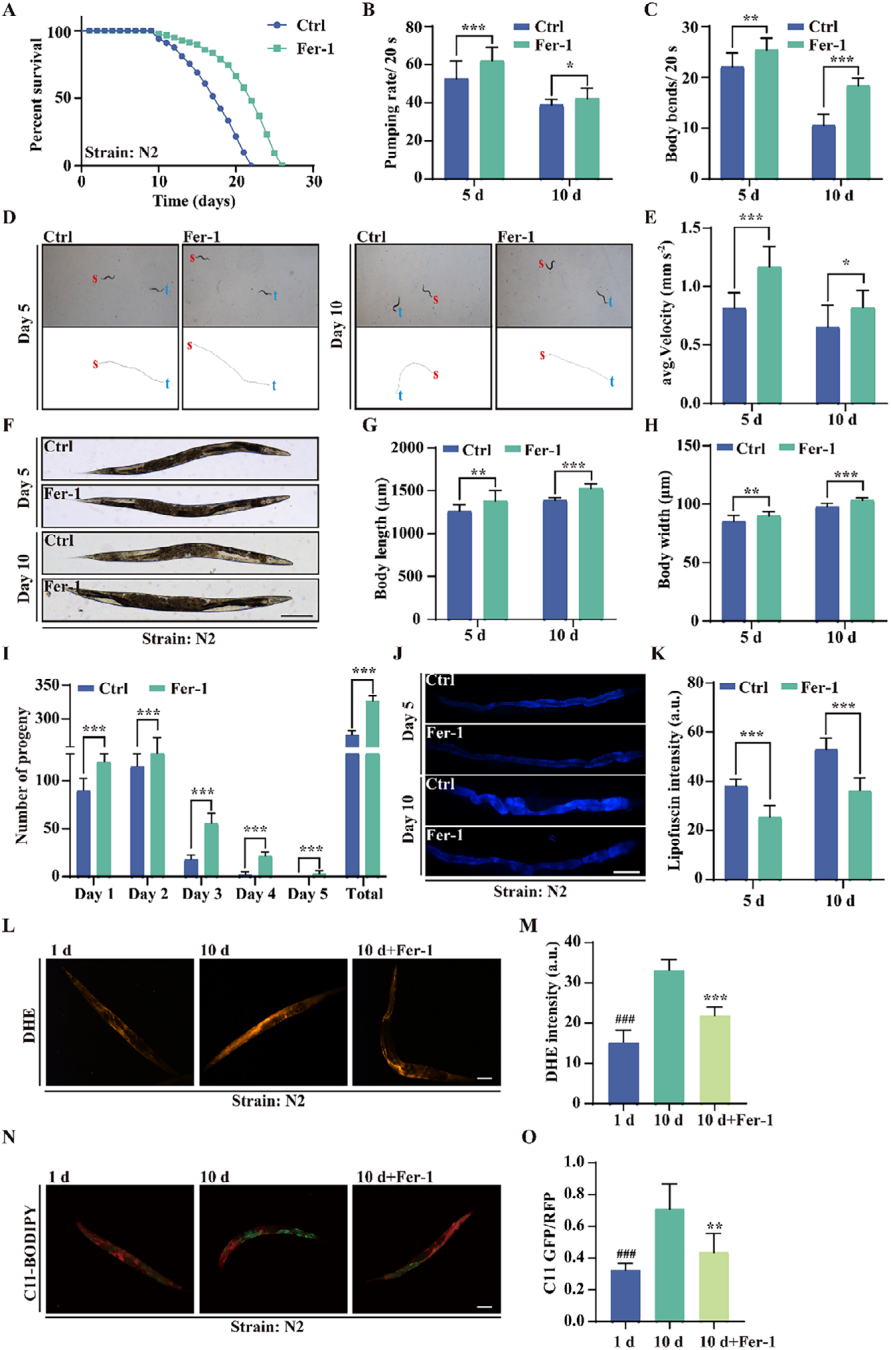
Fer‐1 extends lifespan and promotes healthspan in wild‐type N2 *C. elegans*. A) Survival curves of wild‐type N2 worms treated with Fer‐1. B) Bar graph showing the pharyngeal pumping rate of wild‐type N2 worms over 20 s following Fer‐1 (100 µm) treatment. Data are presented as mean ± SD (*n* = 30 worms); *p < 0.05 and ***p < 0.001. C) Bar graph depicting body bending frequency of N2 worms over 20 s following Fer‐1 treatment. Data are presented as mean ± SD (*n* = 30 worms); **p < 0.01 and ***p < 0.001. D) Representative images of movement trajectories of N2 worms treated with Fer‐1, recorded over 20 s, with “s” marking the start and “t” the end. E) Bar graph illustrating average speed of N2 worms following Fer‐1 treatment. Data are presented as mean ± SD (*n* = 30 worms); *p < 0.05 and ***p < 0.001. F) Representative images showing body length and width of N2 worms treated with Fer‐1. Scale bar: 200 µm. G,H) Bar graphs showing body length and width of N2 worms treated with Fer‐1. Data are presented as mean ± SD (*n* = 30 worms); **p < 0.01 and ***p < 0.001. I) Bar chart showing daily and total progeny of N2 worms treated with Fer‐1. Data are presented as mean ± SD (*n* = 30 worms); ***p < 0.001. J) Representative images showing lipofuscin accumulation in N2 worms treated with Fer‐1. Scale bar: 200 µm. K) Bar graph displaying relative lipofuscin intensity in N2 worms treated with Fer‐1. Data are presented as mean ± SD (*n* = 20 worms); ***p < 0.001. L) Representative images showing intracellular ROS accumulation, labeled with the DHE fluorescent dye in N2 worms treated with Fer‐1. Scale bar: 200 µm. M) Bar graph quantifying the DHE intensity of N2 worms treated with Fer‐1. Data are presented as mean ± SD (*n* = 20 worms); ***p < 0.001. # indicates a significant difference between 1 d and 10 d groups. N) Representative images showing lipid peroxide accumulation labeled with the C11‐BODIPY581/591 fluorescent dye in N2 worms treated with Fer‐1. Scale bar: 200 µm. O) Bar graph quantifying the C11 GFP/RFP ratio in N2 worms treated with Fer‐1. Data are presented as mean ± SD (*n* = 20 worms); **p < 0.01 and ***p < 0.001. # indicates a significant difference between 1 d and 10 d groups.

To assess the impact of ferroptosis inhibition on aging in vivo, we utilized the well‐established *C. elegans* model. Our results showed that Fer‐1 treatment significantly extended the lifespan of wild‐type N2 worms by up to 18.18% (**Figure** **4**A). Given the link between aging and muscle degeneration, we evaluated behavioral changes in Fer‐1‐treated worms at days 5 and 10 of adulthood. Fer‐1 treatment notably improved pharyngeal pumping rates, body bends, maximum motion trajectory, and average speed (Figure 4B–E; Video S1, Supporting information), suggesting a protective effect against age‐related muscle decline. In addition, Fer‐1 increased body length and width at both days 5 and day 10 compared to untreated controls (Figure 4F–H), further indicating enhanced physical fitness. These findings align with previous reports showing that ferroptosis inhibitors, such as Lip‐1 and salicylaldehyde isonicotinoyl hydrazone (SIH), extend lifespan and improve fitness in worms.^[^ ^18a^ ^]^ However, unlike Lip‐1 and SIH, which exhibited neutral or negative effects on reproductive output, Fer‐1 significantly enhanced both daily and total reproductive output (Figure 4I). To further investigate aging‐related changes, we measured lipofuscin accumulation, a marker of cellular senescence and aging.^[^ ^19^ ^]^ As expected, lipofuscin levels increased with age, but Fer‐1 treatment significantly reduced its accumulation at both days 5 and 10 (Figure 4J,K), suggesting a delay in aging progression. These findings demonstrate that Fer‐1 not only extends lifespan but also improves healthspan in *C. elegans*. Next, we examined whether Fer‐1 inhibits ferroptosis in *C. elegans*. DHE and C11‐BODIPY staining revealed a significant increase in ROS and lipid peroxidation by day 10 compared to day 1, indicating that ferroptosis intensifies with age. However, Fer‐1 treatment significantly reduced both ROS levels and lipid peroxidation on day 10 (Figure 4L‐O). Together, these results suggest that ferroptosis plays a central role in aging in *C. elegans*, and Fer‐1 effectively mitigates this process, thereby enhancing both lifespan and healthspan.

### 2.5 Fer‐1 Alleviates Behavioral Deficits and Tissue Injury in D‐Gal‐Induced Premature Aging Mice

**Figure 5:**
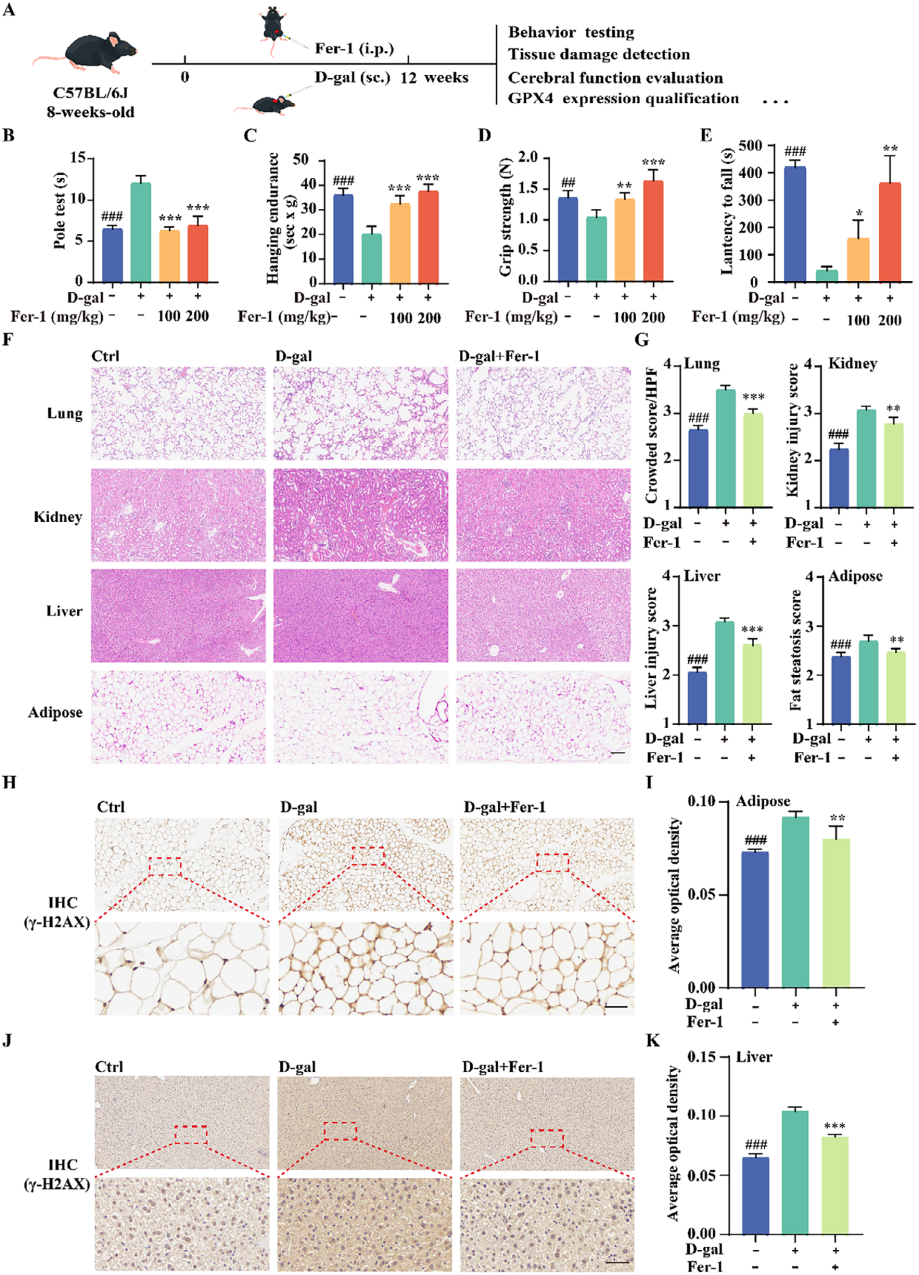
Fer‐1 alleviates behavioral deficits and tissue injury in D‐gal‐induced premature aging mice. A) Schematic representation of experimental design. B–E) Behavioral tests in 8‐week‐old C57BL/6J mice subjected to D‐gal‐induced premature aging and treated with vehicle or Fer‐1 (100 or 200 mg kg^−1^). Assessments include the pole test (B), hanging endurance (C), grip strength (D), and constant speed test (E), conducted 2 weeks post‐treatment. Data are presented as mean ± SD (*n* = 3 independent animals); *p < 0.05, **p < 0.01, and ***p < 0.001. # indicates a significant difference between the control and D‐gal groups. F,G) H&E staining of lungs, kidney, liver, and adipose tissues to observe pathological tissue damage. Bar graphs indicate the pathological scoring. Data are presented as mean ± SD (*n* = 3 independent animals); **p < 0.01 and ***p < 0.001. # indicates a significant difference between the control and D‐gal groups. Scale bar: 200 µm. H,I) Representative IHC staining images of the DNA damage marker γ‐H2AX in adipose tissue. Quantification of the average optical density was performed using ImageJ. Data are presented as mean ± SD (*n* = 3 independent animals); **p < 0.01 and ***p < 0.001. # indicates a significant difference between the control and D‐gal groups. Scale bar: 50 µm. J,K) Representative IHC staining images of the DNA damage marker γ‐H2AX in liver tissue. Quantification of the average optical density was performed using ImageJ. Data are presented as mean ± SD (*n* = 3 independent animals); ***p < 0.001. # indicates a significant difference between the control and D‐gal groups. Scale bar: 50 µm.

To further evaluate the potential of ferroptosis inhibition in delaying aging in vivo, we utilized a D‐gal‐induced premature aging model in C57BL/6 mice. This model mimics key features of natural aging, including cognitive decline, tissue damage, and skin aging, making it a suitable system for assessing anti‐aging interventions.^[^ ^20^ ^]^ In this study, D‐gal‐treated C57BL/6 mice received daily intraperitoneal injections of Fer‐1 at 100 or 200 mg kg^−1^ for 12 weeks (**Figure** **5**A). Long‐term Fer‐1 treatment had no significant effect on body weight in either sex (Figure S3, Supporting information). To assess neuromuscular function, we conducted ladder climbing, hanging endurance, grip strength, and fall latency tests. Fer‐1 treatment significantly mitigated the D‐gal‐induced decline in motor function (Figure 5B–E), indicating that it preserves muscle strength and coordination in prematurely aging mice. Next, we examined the protective effects of Fer‐1 on tissue integrity using H&E staining of the lung, kidney, liver, and adipose tissues. D‐gal treatment caused substantial tissue damage, reflected by elevated pathological scores across all tissues. However, Fer‐1 treatment significantly reduced tissue damage (Figure 5F,G), suggesting cytoprotective effects. Oxidative stress in D‐gal‐treated mice has been linked to DNA damage.^[^ ^21^ ^]^ To determine whether Fer‐1 mitigates oxidative DNA damage, we performed IHC staining for γ‐H2AX, a marker of DNA damage. D‐gal‐treated mice exhibited increased γ‐H2AX staining in adipose tissue and liver, indicating elevated DNA damage. Fer‐1 treatment significantly reduced γ‐H2AX staining intensity, suggesting enhanced DNA repair (Figure 5H–K). Together, these findings demonstrate that Fer‐1 enhances behavioral performance and tissue integrity in D‐gal‐induced prematurely aging mice, further supporting its anti‐aging potential.

### 2.6 Fer‐1 Alleviates Tissue Aging and Increases GPX4 Expression in D‐Gal‐Induced Premature Aging Mice

**Figure 6:**
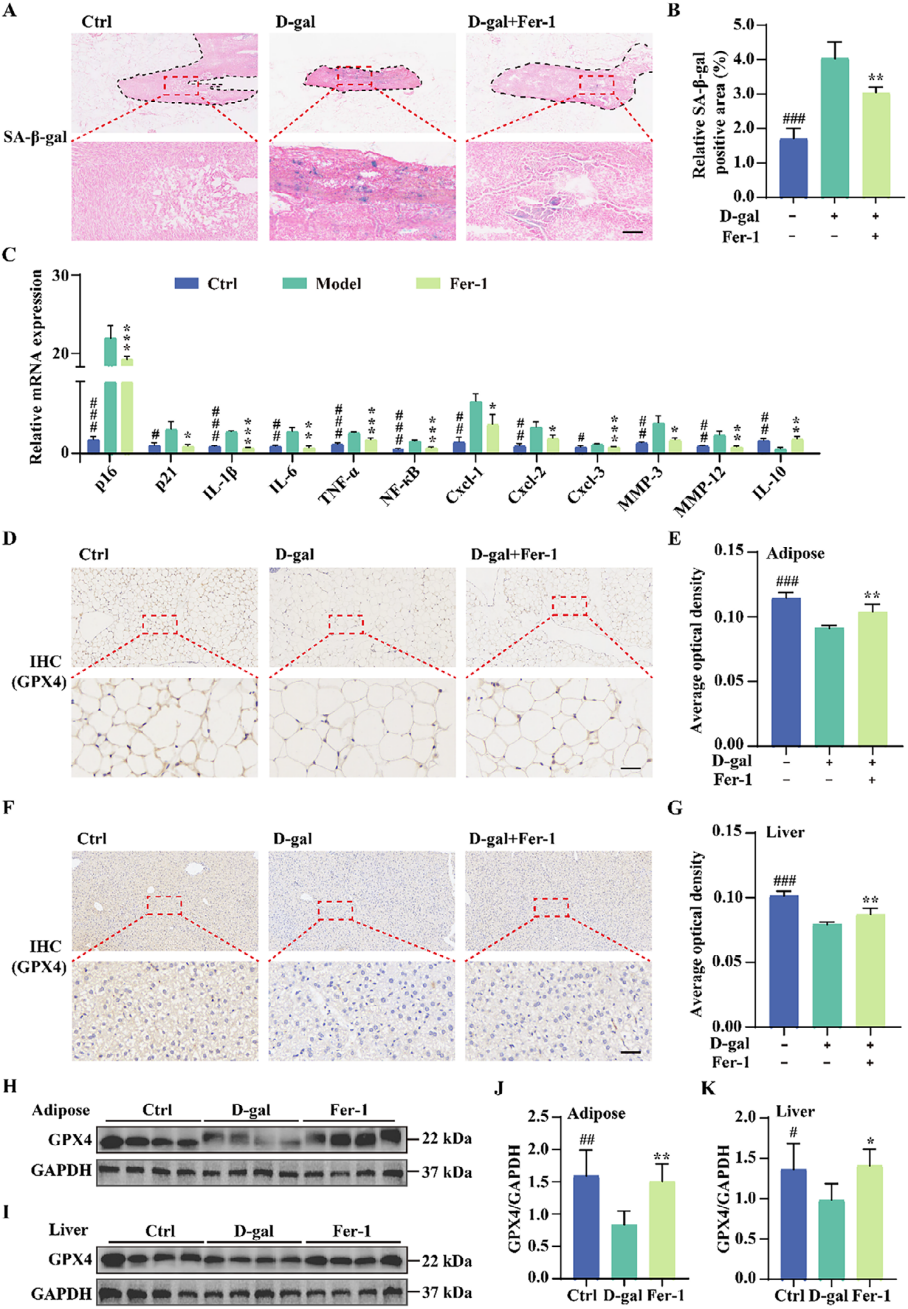
Fer‐1 mitigates tissue aging and enhances GPX4 expression in D‐gal‐induced premature aging mice. A,B) Representative images show SA‐β‐gal‐positive staining in the reproductive glands connected to the epididymal adipose tissue of D‐gal‐induced mice treated with 100 mg kg^−1^ Fer‐1. The dotted circle highlights the reproductive glands; while, the outer dotted circle indicates the epididymal adipose tissue. The bar graph presents the quantification of the positive staining areas. Data are presented as ± SD (*n* = 3 independent animals); **p < 0.01 and ***p < 0.001. # indicates a significant difference between the control and D‐gal groups. Scale bar: 50 µm. C) Bar graph quantifying mRNA levels of p16, p21, and SASP in the adipose tissue of D‐gal‐induced mice treated with 100 mg kg^−1^ Fer‐1. Data are presented as ± SD (*n* = 3 independent animals); *p < 0.05, **p < 0.01, and ***p < 0.001. # indicates a significant difference between the control and D‐gal groups. D,E) IHC staining of GPX4 in adipose tissue. Quantification of average optical density was performed using ImageJ. Data are presented as ± SD (*n* = 3 independent animals); **p < 0.01 and ***p < 0.001. # indicates a significant difference between the control and D‐gal groups. Scale bar: 50 µm. F,G) IHC staining of GPX4 in liver tissue. Quantification was performed using ImageJ. Data are presented as ± SD (*n* = 3 independent animals); **p < 0.01 and ***p < 0.001. # indicates a significant difference between the control and D‐gal groups. Scale bar: 50 µm. H–K) Western blot analysis of GPX4 protein levels in adipose and liver tissues of D‐gal‐induced premature aging mice treated with Fer‐1. Bar graphs quantify GPX4 protein expression. Data are presented as mean ± SD (*n* = 4 independent animals); *p < 0.05 and **p < 0.01. # indicates a significant difference between the control and D‐gal groups.

Aging is closely linked to cellular senescence, which accelerates tissue degeneration.^[^ ^22^ ^]^ To evaluate the effect of Fer‐1 on cellular senescence in vivo, we performed SA‐β‐gal staining on multiple tissues. D‐gal‐treated mice exhibited significant SA‐β‐gal staining in reproductive glands adjacent to epididymal adipose tissue, indicating pronounced senescence, which was markedly reduced by Fer‐1 treatment (**Figure** **6**A,B). In contrast, liver and brain tissues showed minimal staining (Figure S4, Supporting information), suggesting that while behavioral and tissue damage were evident, senescence was not fully manifested in these organs. To further investigate, we conducted qRT‐PCR on adipose tissue, an early responder in age‐related changes that drives systemic aging.^[^ ^23^ ^]^ Fer‐1 significantly reduced mRNA levels of *p16*, *p21*, and key SASP markers, including *IL‐1β*, *IL‐6*, *TNF‐α*, *NF‐κB*, *Cxcl‐1*, *Cxcl‐2*, *Mmp‐3*, and *Mmp‐12*; while, upregulating the anti‐inflammatory factor *IL‐10* (Figure 6C). These results suggest that Fer‐1 mitigates D‐gal‐induced senescence and inflammation. Next, we assessed ferroptosis by examining GPX4 expression, a key ferroptosis inhibitor. IHC staining revealed a significant decline in GPX4 levels in the liver and adipose tissues of D‐gal‐treated mice, which was reversed by Fer‐1 (Figure 6D–G). Western blot analysis further confirmed GPX4 upregulation following Fer‐1 treatment (Figure 6H–K). In conclusion, these findings suggest that Fer‐1 alleviates tissue aging by enhancing GPX4 expression, inhibiting ferroptosis, and reducing senescence‐associated inflammation in D‐gal‐induced premature aging mice.

### 2.7 Fer‐1 Enhances Brain Function in D‐Gal‐Induced Premature Aging Mice

**Figure 7:**
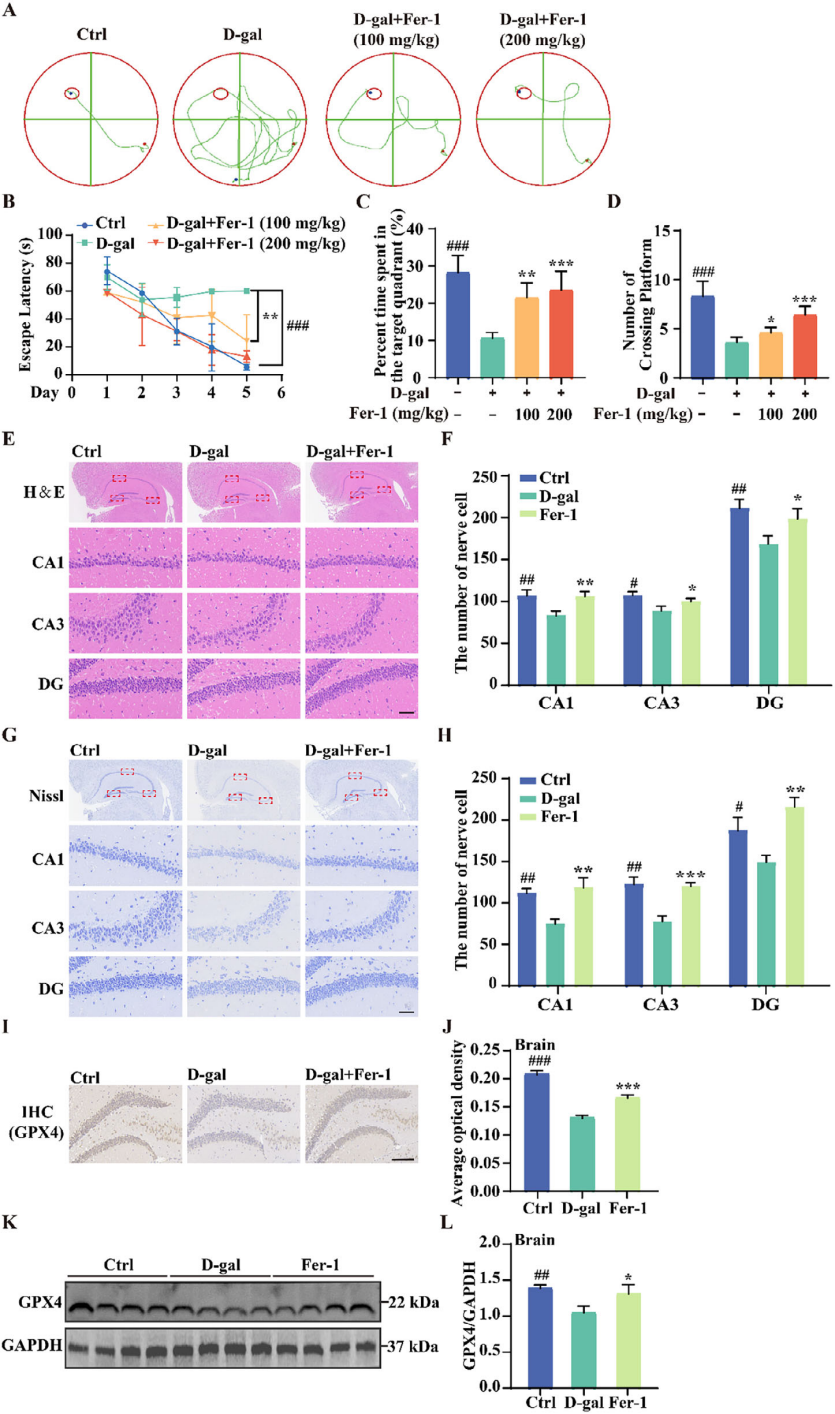
Fer‐1 alleviates brain function decline in D‐gal‐induced premature aging mice. A–D) Morris water maze test assessing spatial recognition memory: representative swim trajectories over five training sessions (A); escape latency recorded from days 1 to 5 (B); Percentage of time spent in the target quadrant during the probe trial (C); and number of platform crossings during the probe trial (D). Data are presented as mean ± SD (*n* = 6 independent animals); *p < 0.05, **p < 0.01, and ***p < 0.001. # indicates a significant difference between the control and D‐gal groups. E,F) H&E staining of the hippocampal region (CA1, CA3, and DG subregions). The bar graph illustrates the average number of nerve cells in these areas. Data are presented as mean ± SD (*n* = 3 independent animals); *p < 0.05 and **p < 0.01. # indicates a significant difference between the control and D‐gal groups. Scale bar: 50 µm. G,H) Nissl staining of the hippocampal region (CA1, CA3, and DG subregions). The bar graph shows the average number of nerve cells in these areas. Data are presented as mean ± SD (*n* = 3 independent animals); *p < 0.05, **p < 0.01, and ***p < 0.001. # indicates a significant difference between the control and D‐gal groups. Scale bar: 50 µm. I,J) IHC staining of GPX4 protein levels in hippocampal tissue. The bar graph quantifies GPX4 expression levels. Data are presented as mean ± SD (*n* = 6 independent animals); ***p < 0.001. # indicates a significant difference between the control and D‐gal groups. Scale bar: 100 µm. K,L) Western blot analysis of GPX4 protein levels in brain tissues. The bar graph quantifies GPX4 expression levels. Data are presented as mean ± SD (*n* = 4 independent animals); *p < 0.05 and **p < 0.01. # indicates a significant difference between the control and D‐gal groups.

Cognitive decline, characterized by deficits in learning and memory, is a hallmark of cerebral dysfunction closely associated with aging. Chronic administration of D‐gal is known to induce cognitive impairments similar to those observed in age‐related conditions.^[^ ^20^ ^]^ This study explored the effects of Fer‐1 on brain function in a D‐gal‐induced premature aging mouse model by assessing its impact on cognitive performance using the Morris water maze. The experiment consisted of two phases: training and testing. During training, mice underwent four trials per day for 5 days, with escape latency (time in seconds) recorded for locating the hidden platform. In the testing phase, the platform was removed, and we measured the percentage of time spent in the target quadrant and the number of platform crossings. The results revealed a significant reduction in escape latency in the Fer‐1‐treated group, along with marked increases in both the time spent in the target quadrant and the number of platform crossings (**Figure** **7**A–D), indicating that Fer‐1 effectively mitigated D‐gal‐induced cognitive decline.

Given the hippocampus's vital role in learning, memory, and spatial navigation,^[^ ^24^ ^]^ we conducted further analyses on hippocampal tissue. H&E staining was used to evaluate overall morphology; while, Nissl staining assessed neuronal health by examining cell body size and structure. H&E staining showed that the hippocampal structure in Fer‐1‐treated mice was more intact, with a significant reduction in tissue damage (Figure 7E,F). Nissl staining indicated that neurons in the Fer‐1 group were healthier, displaying normal cell body size and morphology (Figure 7G,H). In addition, IHC staining and Western blot analyses revealed a significant decrease in GPX4 expression in the hippocampal tissue of D‐gal‐treated mice, which was notably elevated by Fer‐1 treatment (Figure 7I–L). These findings suggest that Fer‐1 enhances brain function and preserves the structural and functional integrity of brain tissue in D‐gal‐induced premature aging mice, potentially through increased GPX4 expression.

### 2.8 Fer‐1 Promotes Healthspan in Naturally Aged Mice

**Figure 8:**
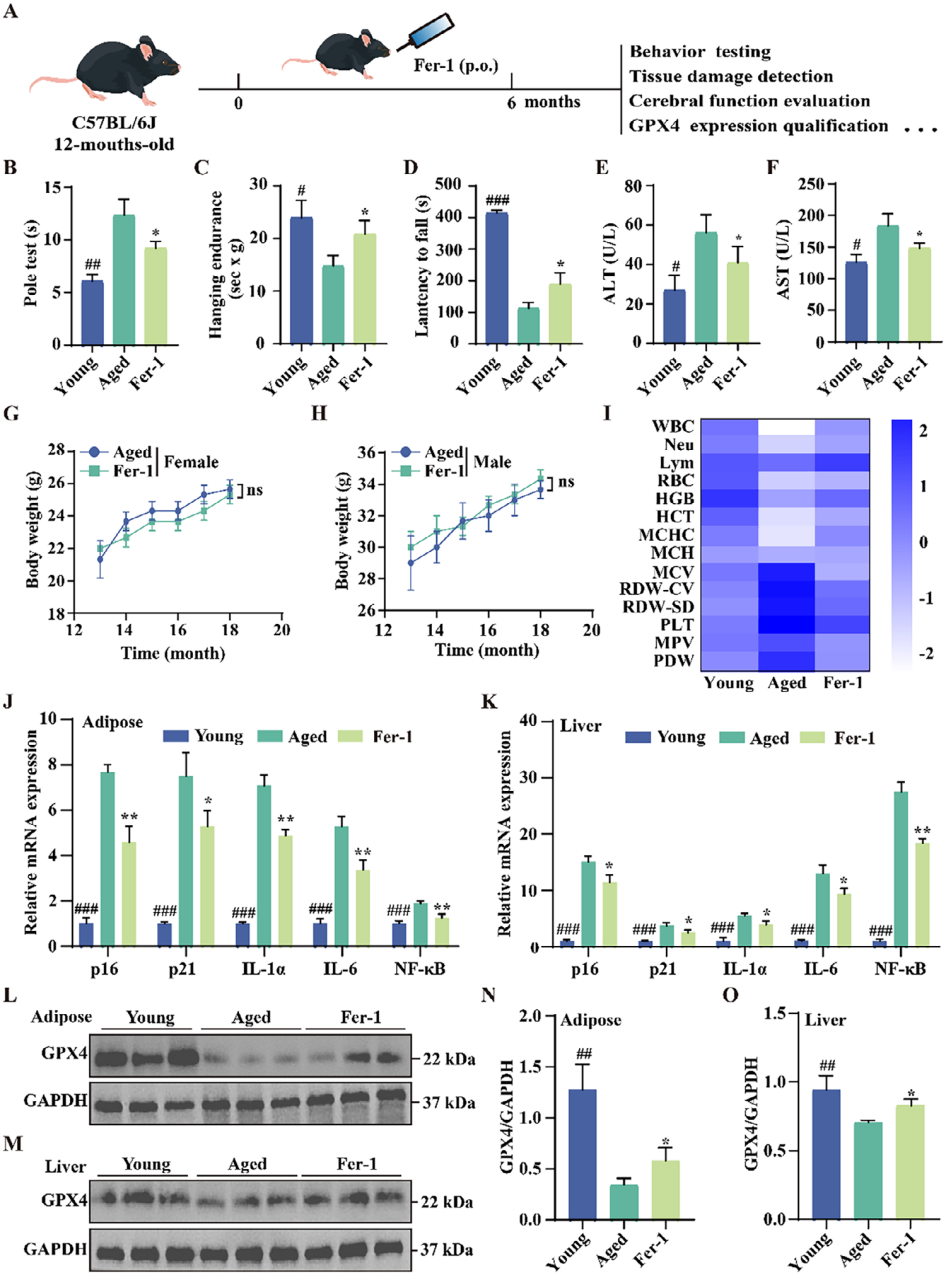
Fer‐1 promotes healthspan in naturally aged mice. A) Schematic representation of experimental design. B–D) Behavioral tests in naturally aged C57BL/6J mice treated with Fer‐1 (100 µm in drinking water), including the pole test (B), hanging endurance test (C), and constant speed test (D). The young group consists of 6‐month‐old C57BL/6J mice. Tests were conducted 2 weeks after the final treatment. Data are presented as mean ± SD (*n* = 6 independent animals); *p < 0.05, **p < 0.01, and ***p < 0.001. # indicates a significant difference between the control and D‐gal groups. E,F) Serum levels of ALT and AST in Fer‐1‐treated naturally aged mice. Data are presented as mean ± SD (*n* = 3 independent animals); *p < 0.05. # indicates a significant difference between the control and D‐gal groups. G,H) Body weight changes in male and female naturally aged C57BL/6J mice treated with Fer‐1. Data are presented as mean ± SD (*n* = 6 mice per group). I) Heatmap of differential hematological parameters in young, old, and Fer‐1‐treated mice. Data are presented as mean ± SD (*n* = 3 mice per group). J,K) mRNA expression analysis of p16, p21, and SASP‐related genes in liver and adipose tissues of naturally aged mice treated with Fer‐1. Data are presented as mean ± SD (*n* = 3 independent animals); *p < 0.05, **p < 0.01, and ***p < 0.001. # indicates a significant difference between the control and D‐gal groups. L–O) Western blot analysis of GPX4 protein levels in adipose and liver tissues of naturally aged mice treated with Fer‐1. Bar graphs quantify GPX4 expression levels. Data are presented as mean ± SD (*n* = 3 independent animals); *p < 0.05 and **p < 0.01. # indicates a significant difference between the control and D‐gal groups.

Building on evidence that Fer‐1 can reverse premature aging in D‐gal‐induced mice, we extended our investigation to assess its effects on healthspan in naturally aged mice. We hypothesized that inhibiting ferroptosis during the aging process could enhance healthspan. To test this, 12‐month‐old C57BL/6 mice were administered Fer‐1 (100 µm) in their drinking water for 6 months (**Figure** **8**A). We evaluated motor abilities and muscle endurance through a series of behavioral tests, including the pole test, hanging endurance test, and latency‐to‐fall test. Aged mice (18 months) exhibited significant declines in these functions compared to young mice (6 months); however, Fer‐1 treatment resulted in marked improvements (Figure 8B–D), indicating that Fer‐1 promotes neuromuscular health and enhances age‐related motor function.

To evaluate the protective effects of Fer‐1 on vital organs and assess potential long‐term toxicity, we monitored liver function, body weight, and tissue integrity. Long‐term administration of Fer‐1 did not harm liver function; rather, it protected against age‐related damage, as evidenced by improved serum levels of alanine aminotransferase (ALT) and aspartate aminotransferase (AST) (Figure 8E,F). In addition, Fer‐1 administration had no significant impact on body weight in either sex (Figure 8G,H). Histological analysis further revealed that Fer‐1 preserved the structural integrity of various tissues, including the lung, liver, kidney, and adipose tissues (Figure S5, Supporting information). Moreover, hemogram analysis demonstrated that Fer‐1 shifted the blood profile of aged mice toward that of young mice (Figure 8I). Specifically, Fer‐1 restored levels of white blood cells (WBC), neutrophils (Neu), and lymphocytes (Lym), indicating enhanced immune function. Fer‐1 also promoted erythropoiesis and improved blood oxygen transport capacity, as reflected by restored levels of red blood cells (RBC), hemoglobin (HGB), and mean hemoglobin concentration (MCHC), suggesting effective amelioration of anemia. Further, Fer‐1 reduced both platelet count and volume, suggesting inhibited platelet activation and aggregation, which may lower the risk of thrombosis in aged mice. Overall, these results indicate that Fer‐1 promotes the healthspan of aged mice.

Subsequently, qRT‐PCR analysis revealed that Fer‐1 significantly downregulated aging‐related genes, including *p16* and *p21*, as well as SASP markers such as *IL‐1α*, *IL‐6*, and *NF‐κB*, in the adipose and liver tissues of naturally aged mice (Figure 8J,K). These findings underscore Fer‐1's potential in alleviating tissue aging and inflammation. In addition, Western blot analysis showed a significant increase in GPX4 expression in both liver and adipose tissues of Fer‐1‐treated aged mice (Figure 8L–O). Collectively, these results suggest that Fer‐1 promotes healthspan, along with inhibiting ferroptosis, in naturally aged mice.

### 2.9 Fer‐1 Alleviates the Decline of Brain Function in Naturally Aged Mice

**Figure 9:**
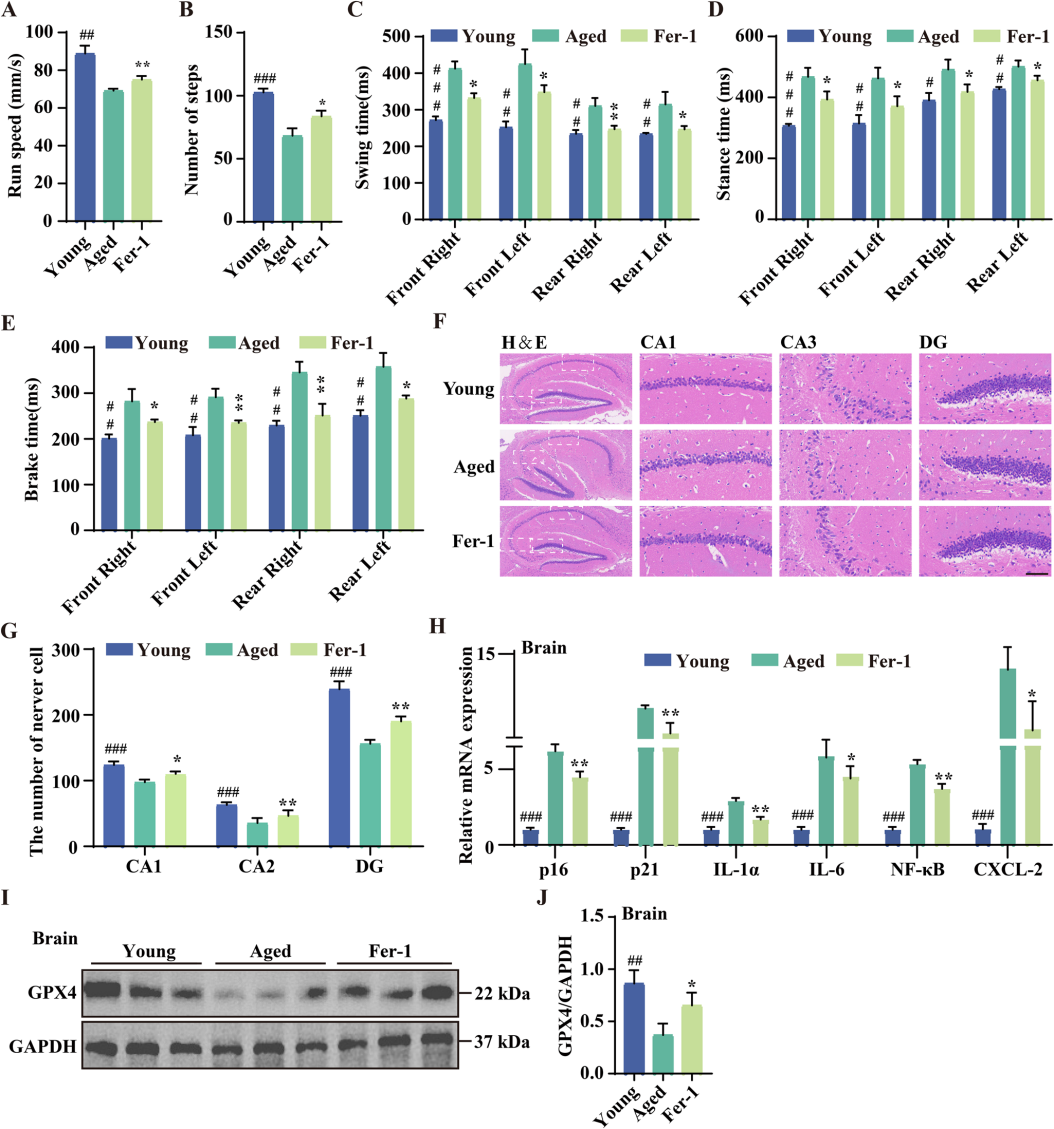
Fer‐1 alleviates brain function decline in naturally aged mice. A–E) Gait analysis metrics: run speed (A), number of steps (B), swing time (C), stance time (D), and brake time (E). Data are presented as mean ± SD (*n* = 6 independent animals); *p < 0.05, **p < 0.01, and ***p < 0.001. # indicates a significant difference between the control and D‐gal groups. F,G) H&E staining of the hippocampal region magnifications of the CA1, CA3, and DG subregions. The bar graph illustrates the average number of nerve cells in these areas. Data are presented as mean ± SD (*n* = 3 independent animals); *p < 0.05, **p < 0.01, and ***p < 0.001. # indicates a significant difference between the control and D‐gal groups. Scale bar: 50 µm. H) mRNA expression analysis of p16, p21, and SASP‐related genes in the brain tissue of naturally aged mice treated with Fer‐1. Data are presented as mean ± SD (*n* = 3 independent animals); *p < 0.05, **p < 0.01, and ***p < 0.001. # indicates a significant difference between the control and D‐gal groups. I,J) Western blot analysis of GPX4 protein levels in brain tissues of naturally aged mice treated with Fer‐1. Bar graphs quantify GPX4 expression levels. Data are presented as mean ± SD (*n* = 3 independent animals); *p < 0.05 and **p < 0.01. # indicates a significant difference between the control and D‐gal groups.

To further explore Fer‐1's potential in mitigating age‐related cognitive decline, we systematically evaluated its effects in naturally aged mice. First, motor function was assessed through gait analysis, measuring parameters such as run speed, step count, swing time, stance time, and brake time—key indicators of coordination and neuromuscular health. The results revealed significant improvements in gait parameters in Fer‐1‐treated mice, including increased speed and enhanced stability (**Figure** **9**A–E), suggesting that Fer‐1 protects against age‐related motor impairments by preserving neuromuscular health. Next, we evaluated the structural integrity of the hippocampus, a critical region for learning and memory, using H&E staining. The hippocampal structure in Fer‐1‐treated mice exhibited improved integrity, with more orderly neuronal arrangements and healthier cell morphology (Figure 9F,G), indicating that Fer‐1 supports cognitive function by maintaining hippocampal health. We also analyzed the expression of aging‐related genes, including *p16* and *p21*, and SASP markers such as *IL‐1α*, *IL‐6*, *NF‐κB*, and *CXCL‐2* in the brains of these mice. Fer‐1 treatment significantly reduced the expression of these markers (Figure 9H), suggesting that Fer‐1 protects brain function by reducing inflammation and slowing the aging process. Finally, Western blot analysis revealed a significant increase in GPX4 expression in the brains of Fer‐1‐treated naturally aged mice (Figure 9I,J), consistent with the results observed in liver and adipose tissues. Collectively, these findings demonstrate that ferroptosis inhibition with Fer‐1 during aging promotes overall health by enhancing motor performance, reducing tissue damage, and preserving brain function in naturally aged mice.

## 3 Discussion

In this study, we conducted a comprehensive, multi‐level investigation using diverse models to elucidate the crucial role of ferroptosis in both cellular and organismal aging. Our findings demonstrate that inhibiting ferroptosis effectively delays aging and enhances healthspan in mammals. These results provide new insights into the ferroptosis‐aging relationship, establishing a strong scientific foundation for developing anti‐aging therapies with broad application and societal impact.

Previous research has identified ferroptosis as a key form of cell death implicated in neurodegenerative diseases,^[^ ^25^ ^]^ cardiovascular disorders,^[^ ^26^ ^]^ and cancer.^[^ ^27^ ^]^ These studies highlight its essential role in disease onset and progression, demonstrating that ferroptosis inhibition can significantly improve outcomes. However, most research has focused on its pathological relevance, largely overlooking its role in natural aging, particularly in cellular senescence, a fundamental driver of age‐related diseases. Our study addressed this gap by providing compelling evidence that ferroptosis significantly influences natural aging. Using D‐gal‐ and DOXO‐induced senescence models, as well as replicative senescence, we observed a progressive increase in lipid peroxidation and ROS levels, accompanied by the downregulation of ferroptosis regulators (GPX4, FTL) and upregulation of ACSL4. These molecular changes indicated ferroptosis activation and underscored its central role in cellular senescence. Further validation using ferroptosis inducers (Erastin, RSL3) demonstrated that ferroptosis accelerated cellular senescence, as indicated by increased β‐galactosidase activity, p16, p21, and SASP factors. Conversely, ferroptosis inhibitors (Fer‐1, Lip) alleviated senescence induced by D‐gal, DOXO, and replicative aging; while, also delaying senescence triggered by Erastin and RSL3. These findings establish ferroptosis as both a driver and regulator of aging, demonstrating that its inhibition effectively delays cellular senescence. Our study systematically links ferroptosis to aging across multiple models, highlighting its potential as a biomarker and therapeutic target. By expanding the scope of ferroptosis research in aging, these insights pave the way for novel anti‐aging interventions targeting this pathway.

Recent studies have shown that ferroptosis inhibition with agents such as Lip and SCH extends lifespan and improves health in *C. elegans*.^[^ ^18a^ ^]^ Our study expands on these findings, demonstrating that another ferroptosis inhibitor, Fer‐1, offers similar benefits. Fer‐1 not only prolongs the lifespan of *C. elegans* but also enhances locomotor activity, increases pharyngeal pumping rate, boosts reproductive output, reduces lipofuscin accumulation, and lowers levels of lipid peroxides and ROS associated with aging. Importantly, we provide crucial evidence that ferroptosis inhibition delays aging and enhances healthspan in mammals. In both D‐gal‐induced and naturally aged mice, Fer‐1 improves motor function, reduces tissue damage and inflammation, and enhances DNA repair. Notably, it exhibits strong neuroprotective effects, improving cognitive function in D‐gal‐induced aging mice (Morris water maze test) and gait in naturally aged mice. In addition, Fer‐1 preserves brain structure and function; while, upregulating GPX4, a key ferroptosis regulator. These findings reinforce the broad anti‐aging potential of ferroptosis inhibitors across species, underscoring their relevance as therapeutic targets. Future research should focus on optimizing their efficacy across different aging stages and species, investigating dose‐response relationships, time‐dependent effects, and combination therapies. Elucidating these mechanisms will advance the clinical application of ferroptosis inhibitors and enhance their potential to extend healthy lifespan.

The safety profile of ferroptosis inhibitors in clinical settings appears promising. In our 6‐month continuous administration study with naturally aged mice, Fer‐1 did not cause significant changes in body weight or apparent tissue damage, including in the liver and kidneys. Instead, Fer‐1 exhibited protective effects against age‐related tissue deterioration, further highlighting its potential as an anti‐aging therapeutic. In addition, Fer‐1 helped normalize hematological parameters, bringing them closer to those of young animals, which may enhance immune function, alleviate anemia, and reduce thrombosis risk. These findings suggest ferroptosis inhibitors hold strong clinical promises for anti‐aging applications. Future studies should focus on developing more effective, low‐toxicity inhibitors, optimizing their pharmacological profiles and rigorously evaluating safety and efficacy across different dosages and administration regimens.

While this study offers new insights into the role of ferroptosis in aging, several critical scientific questions remain unresolved. First, despite well‐documented connections between ferroptosis and aging, its precise role in the aging process requires further investigation. Second, the interactions between ferroptosis and other forms of cell death, such as apoptosis and autophagy, and their combined effects on aging need deeper exploration. Understanding these complex interactions could facilitate the development of more targeted anti‐aging strategies. In addition, validating ferroptosis‐based anti‐aging approaches in more advanced animal models, such as rats or primates, which closely mimic the human aging process, will yield more clinically relevant safety and efficacy data. Finally, investigating the intricate relationships between ferroptosis and other aging mechanisms—especially in conjunction with other anti‐aging interventions—could lead to more effective and comprehensive strategies for slowing aging and extending a healthy lifespan.

**Figure 10:**
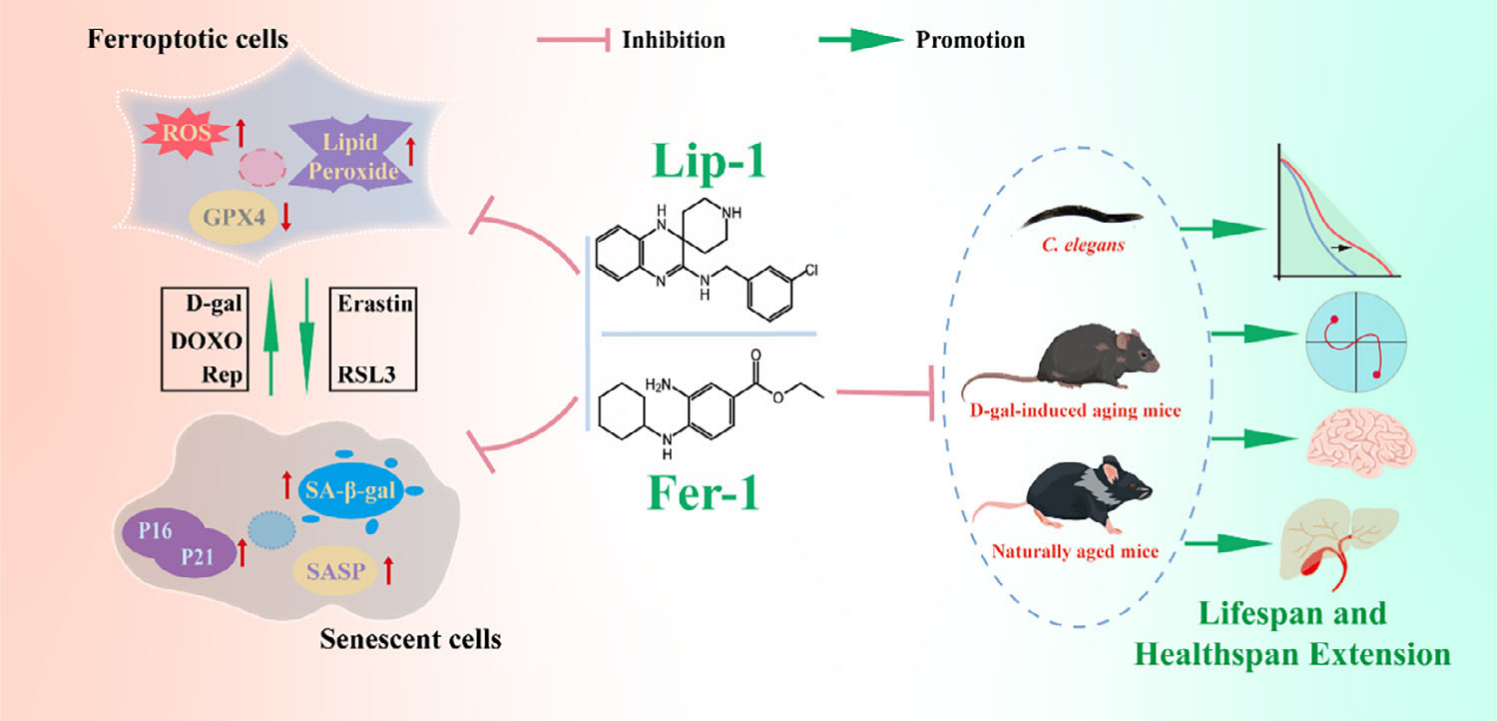
Summary of the study. This study demonstrates that ferroptosis is a key driver of cellular senescence, and its inhibition delays aging and extends healthspan across species. Ferroptosis is exacerbated during D‐gal, DOXO, and replicative‐induced senescence, characterized by lipid peroxidation, oxidative stress, and reduced GPX4. Ferroptosis inducers, such as Erastin and RSL3, accelerate senescence; while, inhibitors such as Lip‐1 and Fer‐1 effectively mitigate it. In vivo, Fer‐1 extends lifespan, improves motor function, preserves tissue integrity, and reduces cognitive decline in *C. elegans*, D‐gal‐induced aging mice, and naturally aged mice by upregulating GPX4 and inhibiting ferroptosis.

In summary, this study employs diverse models and multi‐level approaches to enhance our understanding of ferroptosis in aging, demonstrating that its inhibition not only slows cellular senescence but also provides broader health benefits (**Figure** **10**). These findings establish a strong scientific foundation for ferroptosis inhibitors as anti‐aging therapies, offering new perspectives and strategies to address global aging challenges; while, deepening our understanding of aging mechanisms.

## 4 Experimental Section

### Cell Lines and Culture

Primary human foreskin fibroblast (HFF) cells were kindly provided by Dr. Pei Xu from the Pediatric Surgery Department, Southwest Medical University Affiliated Hospital, Sichuan, China. HFF cells were maintained in Dulbecco's Modified Eagle Medium (DMEM; Gibco, Scotland, UK), supplemented with 10% fetal bovine serum (FBS), 50 µg mL^−1^ streptomycin, and 50 U mL^−1^ penicillin (Invitrogen, Scotland, UK). The cells were cultured at 37 °C in a humidified incubator with 5% CO₂.

### *C. Elegans* Strains and Culture

The wild‐type *C. elegans* strain N2 was obtained from the Caenorhabditis Genetics Center (CGC), University of Minnesota, USA. N2 worms were fed with *Escherichia coli* OP50 and cultured on Nematode Growth Medium (NGM) plates at 20 °C. To synchronize the larvae, gravid nematodes were treated with a bleaching solution containing 0.5 m NaOH and 1% NaClO to release eggs, which were then allowed to hatch at 20 °C for at least 12 h.

### Reagents and Antibodies

Liproxstatin‐1 (Lip‐1, #950455‐15‐9) and Ferrostatin‐1(Fer‐1, #347174‐05‐4) were purchased from Beijing Psaitong Biotechnology Co., Ltd (Beijing. China). D‐galactose (D‐gal, #S11050‐100g) was purchased from Source BioScience Ltd. (Shanghai, China). Doxorubicin hydrochloride (DOXO, #25316‐40‐9) was gained from Shanghai Macklin Biochemical Technology Co., Ltd (Shanghai, China). The antibody against glutathione peroxidase 4 (GPX4, #HY‐P80450) was sourced from MedChemExpress (MCE, NJ, USA). The antibody for acyl‐CoA synthetase long‐chain family member 4 (ACSL4, #22401‐1‐AP) was acquired from Proteintech Group, Inc. The antibody against ferritin light chain (FTL, #A1768) was obtained from ABclonal Technology Co., Ltd (Wuhan, China). Antibodies against cyclin‐dependent kinase inhibitor 2A (CDKN2A, P16, #80772) and cyclin‐dependent kinase inhibitor 1A (CDKN1A, P21, #2947) were purchased from Cell Signaling Technology Inc. (CST, Beverly, MA, USA). The GAPDH antibody (#sc‐47724) was sourced from Santa Cruz Biotechnology, Inc. (Santa Cruz, CA, USA).

### Establishment of Cell Senescence Models

To induce cellular senescence, HFF cells were treated with either 80 and 160 mm D‐gal, or with 80 and 160 nm DOXO for 5 days. After treatment, cells were rinsed three times with phosphate‐buffered saline (PBS) and returned to standard culture conditions. For replicative senescence (Rep), passage‐5 HFF cells were subcultured at a 1:2 or 1:3 ratio until they exhibited characteristic senescent features, including slowed proliferation, increased cell size, and flattened morphology. Rep cells typically emerged after 20–40 passages. Senescence was confirmed in each experiment using SA‐β‐gal staining and morphological assessments.

### SA‐β‐Gal Staining in Cells

SA‐β‐gal activity in HFF cells was assessed using a SA‐β‐gal staining kit (#G1580; Solarbio Biotech Co., Ltd., Beijing, China). Cells were first fixed with 3% formaldehyde solution for 15 min, followed by three washes with PBS, each lasting 3 min. After washing, the SA‐β‐gal staining solution was added, and cells were incubated overnight at 37 °C. For quantitative analysis, random fields were observed, and the percentage of SA‐β‐gal‐positive cells was calculated relative to the total cell count.

### RNA Isolation and Quantitative Real‐Time PCR (qRT‐PCR)

Total RNA was extracted from cells or tissues using TRIzol reagent (R1100; Solarbio, Beijing, China). Reverse transcription was performed using a two‐step cDNA synthesis kit (R323‐01; Vazyme, Nanjing, China). qRT‐PCR was conducted using SYBR Green PCR Master Mix (Q711‐02; Vazyme, Nanjing, China), with GAPDH serving as the internal control for normalization. Each sample was analyzed in triplicate, and the average CT value was used for quantification. Primer sequences are listed in Table S1, Supporting Information.

### *C. elegans* Phenotype Assays

For the lifespan assay, *C. elegans* was cultured on NGM plates supplemented with Fer‐1, and survival was monitored daily. Each group consisted of 100–150 worms, with experiments performed at 20 °C in a temperature‐controlled chamber. Each assay was conducted in triplicate. Feeding activity was assessed by counting pharyngeal pumping events over 20 s under a stereomicroscope (Leica M205FA, Leica Microsystems GmbH, Germany). Each worm was measured three times, and the average calculated. Body bend frequency was determined by counting movements in M9 buffer over 20 s, with each worm undergoing three trials. Movement trajectories were recorded using a stereomicroscope, and speed was analyzed using movement analysis software under controlled conditions. Body length and width were measured on days 5 and 10 of adulthood using ImageJ software (National Institutes of Health, Bethesda, MD, USA), with at least 50 worms per group. Fecundity was evaluated by recording the number of eggs laid daily from day 1 to day 5 of adulthood, with 20–30 worms per group. Each experiment was repeated in triplicate. Fluorescence imaging of lipofuscin accumulation was performed on days 5 and 10 of adulthood, and fluorescence intensity was analyzed using ImageJ software. Detailed descriptions of all methods can be found in the authors previous study.^[^ ^28^ ^]^

### Measurement of ROS Levels in Cells and *C. elegans*

ROS levels were assessed in both HFF cells and nematodes. For HFF cell, cells were incubated with the ROS‐sensitive fluorescent probe DHE (#BN1108‐1ml, BIORIGIN Biotech Co., Ltd.) at 37 °C in a 5% CO₂ environment for 30 min in the dark. After incubation, cells were washed twice with phosphate‐buffered saline (PBS), and six random fields per group were imaged using a fluorescence microscope (Leica DM6B, Leica Microsystems GmbH, Germany). For *C. elegans*, worms were collected in 1.5 mL EP tubes and washed three times with M9 buffer. The fluorescent probe was then added, and samples were incubated at 20 °C for 1 h in the dark, with gentle flicking of the tube wall every 5 min. After staining, nematodes were washed three times with M9 buffer and imaged using a fluorescence microscope. Fluorescence intensity was analyzed using ImageJ software.

### Measurement of C11 BODIPY 581/591 Levels in Cells and *C. elegans*

Lipid peroxidation was evaluated using C11 BODIPY 581/591 (#27086, Cayman Chemical Company, Inc., CA, USA) in both cells and nematodes. For cell experiments, the dye was diluted 1:1000 in serum‐free, antibiotic‐free culture medium and added (100 µL per well) to a 96‐well plate. Cells were incubated at 37 °C in a 5% CO₂ environment for 30 min in the dark, followed by two PBS washes. Six random fields per group were imaged using a fluorescence microscope. For nematode experiments, worms were collected in 1.5 mL EP tubes, washed three times with M9 buffer, and incubated with C11 BODIPY 581/591 (1:100 dilution) at 20 °C for 1 h in the dark, with gentle flicking every 5 min. After staining, nematodes were washed three times with M9 buffer and imaged using a fluorescence microscope. Fluorescence intensity was analyzed using ImageJ software.

### Establishment of D‐Gal‐Induced Premature Aging Mice

Eight‐week‐old specific pathogen‐free (SPF) C57BL/6J mice were housed under controlled conditions (23 °C ± 2 °C, 40–75% humidity, 12‐h light/dark cycle) with ad libitum access to food and water. After a 1‐week acclimation period, the mice were randomly divided into four groups (ten mice per group, with balanced sex distribution): 1) Normal control group (treated with normal saline), 2) premature aging model group (administered 200 mg kg^−1^ D‐gal), 3) low‐dose Fer‐1 group (co‐treated with 200 mg kg^−1^ D‐gal and 100 mg kg^−1^ Fer‐1), and 4) high‐dose Fer‐1 group (co‐treated with 200 mg kg^−1^ D‐gal and 200 mg kg^−1^ Fer‐1). The aging induction and treatment continued for 12 weeks. At the end of the study, mice were anesthetized with 150 mg kg^−1^ sodium pentobarbital and euthanized. Tissues were then collected for subsequent analyses, including RNA extraction, Western blotting, immunohistochemistry (IHC), and hematoxylin and eosin (H&E) staining. All procedures followed the guidelines of the Animal Ethics Committee of Southwest Medical University (Approval No.: 20221107‐010, November 7, 2022).

### Physical Function Tests of Mice

The grip strength of the mice was assessed using a grip strength meter (China Sinopec Dichuang Technology Development Co., Ltd.), with each mouse undergoing three trials. Balance and coordination were evaluated using the balance beam test, where mice traversed a horizontal beam (2 cm in diameter, 50 cm in length) after four acclimation trials at each stage. During testing, four trials were conducted, recording the time taken to cross the beam and any instances of falling. Endurance was measured by suspending mice from a 2 mm thick metal wire, 40 cm above a cushioned surface, with the hanging duration recorded. The hanging times were adjusted for body weight by multiplying the duration (in seconds) by the mouse's weight (in grams), and the average of three trials was calculated. Motor coordination and endurance were further assessed using the rotarod test, where mice were trained for three consecutive days at 4, 6, and 8 RPM for 120 s per session. On the test day, the rotarod speed started at 4 RPM, gradually increased to 10 RPM over 10 s, and was maintained at that speed for 10 min during the constant‐speed phase.

### H&E Staining and Pathological Scoring of Tissues

For H&E staining, lung, kidney, liver, and adipose tissues (epididymal adipose tissue) from C57BL/6J mice were collected and immediately fixed in 10% neutral buffered formalin for 24–48 h. The tissues were then dehydrated through a graded ethanol series, cleared in xylene, and embedded in paraffin. Thin sections (3–5 µm) were cut using a microtome and mounted on glass slides. After deparaffinization in xylene and rehydration through alcohol gradients, the sections were stained with hematoxylin to highlight nuclei, followed by eosin to stain the cytoplasm. The slides were subsequently dehydrated, cleared, and coverslipped for microscopic examination. For lung injury assessment, the extent of crowded areas—defined as regions of thickened septa in the lung parenchyma associated with partial or complete alveolar collapse—was measured in a blinded manner, following established protocols.^[^ ^29^ ^]^ Kidney injury scores were determined by evaluating tubular necrosis, loss of brush border, cast formation, and tubular dilatation, also in a blinded manner, as previously described.^[^ ^30^ ^]^ Liver injury was assessed by the degree of hepatic fibrosis, with blind measurements conducted according to previously described methods.^[^ ^31^ ^]^ For the assessment of adipocytes, image analysis software was utilized to measure the area of each adipocyte and calculate the average size. This method effectively assessed changes in adipocyte size, reflecting pathological conditions such as metabolic dysfunction and inflammatory responses.^[^ ^32^ ^]^

### IHC Staining of Mouse Tissues

IHC staining was conducted to assess protein expression in mouse tissue samples. Tissue sections (4–6 µm thick) were prepared from paraffin‐embedded or frozen tissues. Paraffin sections underwent deparaffinization and rehydration, whereas frozen sections were processed directly. For antigen retrieval, sections were heated in citrate or Tris‐EDTA buffer to enhance antigen exposure. Endogenous peroxidase activity was blocked using 3% hydrogen peroxide (H₂O₂) to minimize background interference. To reduce non‐specific binding, sections were incubated with 5% bovine serum albumin (BSA) or normal serum. Next, sections were incubated with the primary antibody for 1–2 h at room temperature or overnight at 4 °C. After thorough washing, an enzyme‐conjugated secondary antibody was applied for 30–60 min to amplify the signal. Protein detection was performed using diaminobenzidine (DAB), generating a brown precipitate at positive sites. Hematoxylin counterstaining was used to visualize nuclei and enhance tissue morphology. Finally, sections were dehydrated through a graded ethanol series, cleared with xylene, and mounted for long‐term preservation.

### Establishment of Naturally Aged Mice

Twelve‐month‐old SPF C57BL/6J mice were housed in controlled conditions (23 °C ± 2 °C, 40–75% humidity, 12h light/dark cycle) with ad libitum access to food and water. Mice were randomly divided into two groups (six mice per group, with equal sex distribution): 1) a control group receiving normal drinking water and 2) a treatment group receiving drinking water supplemented with Fer‐1 (100 µm). The regimen was administered for 6 months. After the treatment period, mice were used to perform gait analysis. After that, mice were anesthetized with 150 mg kg^−1^ sodium pentobarbital and euthanized. Tissues were collected for analysis, including RNA and protein extraction, and paraffin embedding for H&E staining. All procedures adhered to the guidelines of the Animal Ethics Committee of Southwest Medical University (Approval No.: 20221107‐010, November 7, 2022).

### Gait Analysis of Naturally Aged Mice

Gait patterns of the mice were analyzed using the GaitScan system (Mouse Specifics Inc.). Prior to testing, the mice were trained on an adaptive treadmill to ensure consistent walking behavior. During the testing phase, each mouse was placed on a transparent treadmill set at 5 cm s^−1^ (adjustable as needed); while, a camera positioned beneath the treadmill captured images of their gait. Key gait parameters, including speed, step count, swing time, stance time, and braking time, were extracted from these images. All measurements were conducted in a controlled environment to minimize behavioral variability. Each mouse underwent three independent trials, and the average values of the parameters were calculated and analyzed using specialized gait analysis software, with data standardized to account for individual differences.

### Data Analysis and Statistics

Statistical analyses were performed using data from a minimum of three independent experiments. Results are presented as mean ± standard deviation (SD) or standard error of the mean (SEM) for qRT‐PCR data. Survival curves from lifespan assays were evaluated with the Kaplan–Meier method, and *p*‐values were calculated using the log‐rank test. For all other assays, two‐tailed *t*‐tests were applied to obtain *p*‐values. Data analysis utilized SPSS version 19 (IBM, Armonk, USA) and GraphPad Prism 9 (San Diego, CA, USA). Statistical significance was set at *p* < 0.05.

### Ethics Approval Statement

All procedures adhered to the guidelines of the Animal Ethics Committee of Southwest Medical University (Approval No.: 20221107‐010, November 7, 2022).

## Conflict of Interest

The authors declare no conflict of interest.

## Author Contributions

A.‐G.W., J.‐M.W., and X.‐G.Z. supervised and designed the experiments. H.‐J.F. and X.‐Y.Z. conducted experiments. Q.Q., Q.‐Z.W., S.‐Y.L., Y.‐F.Z., Y‐P.L., J.‐M.Z., H.C., and F.‐H.H. collected and processed the data. D.‐L.Q., L.Y., L.W., and A.‐G.W. participated in discussions for the paper; H.‐J.F. and X.‐G.Z. wrote the paper.

## Supporting information



Supporting Information

Supplemental Video 1

## Data Availability

The data that support the findings of this study are available in the supplementary material of this article.
