# Supplementary material for: Inhibition of Ferroptosis Delays Aging and Extends Healthspan Across Multiple Species
Source: Adv Sci (Weinh). 2025 Mar 31;12(22):2416559. doi: 10.1002/advs.202416559 (PMC12165029; doi:10.1002/advs.202416559)
Supplement: Supplementary file 1 — Supporting Information [file ADVS-12-2416559-s002.docx]

**Supplementary Material**

**Supplementary Figure Legends**


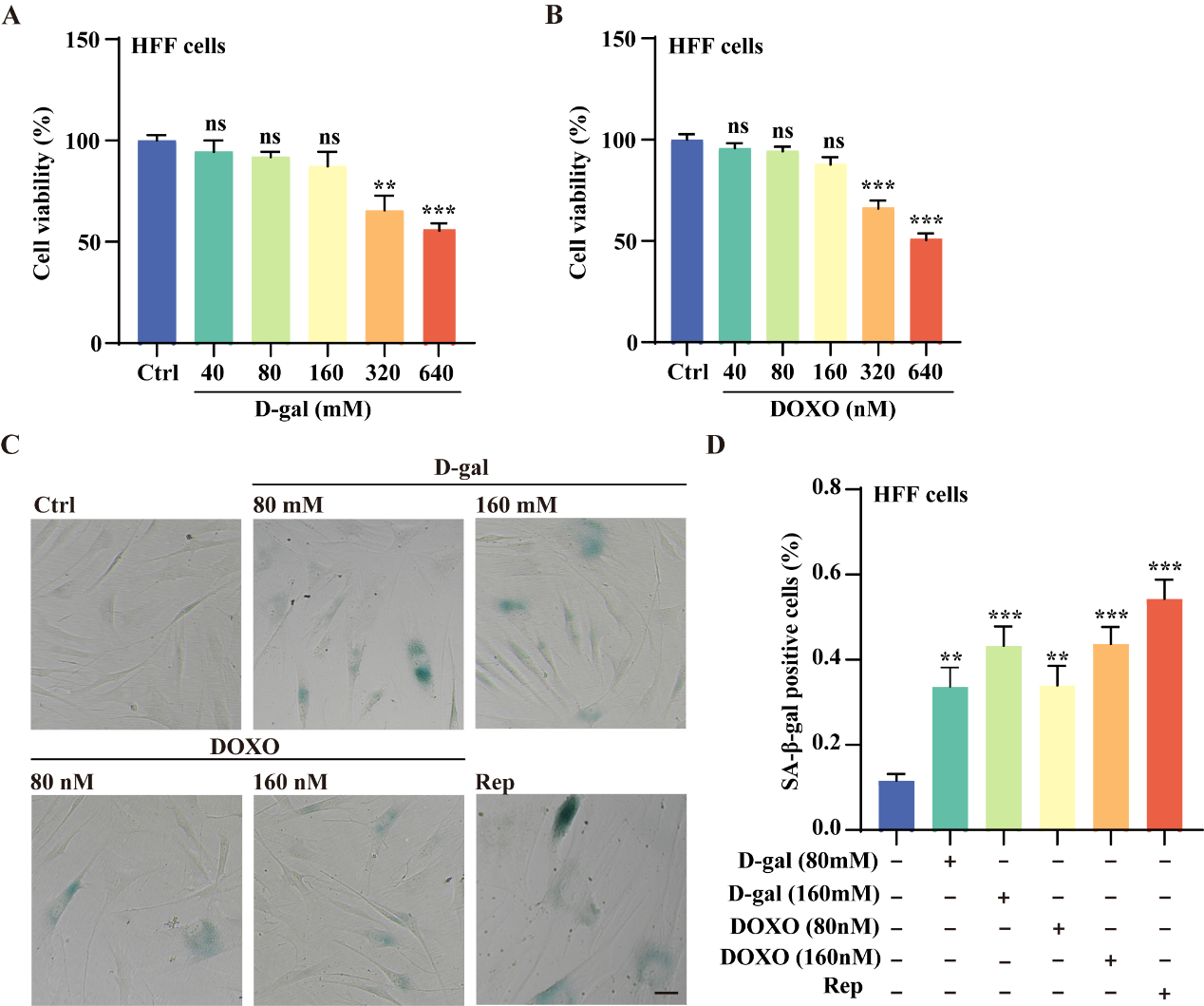


**Figure S1. Establishment of three senescence models in HFF cells. (A-B)** Bar graph showing the viability of HFF cells treated with various concentrations of D-gal and DOXO. Data are presented as mean ± SD (n= 3 independent experiments); ns *p* > 0.05，***p* < 0.01, ****p* < 0.001. **(C)** SA-β-gal staining in HFF cells treated with D-gal (80–160 mM) and DOXO (80–160 nM), as well as in replicative senescent HFF cells. Representative images show control and senescent HFF cells. Scale bars: 25 µm. **(D)** Bar graph depicting the percentage of SA-β-gal-positive cells in (C). Data are presented as mean ± SD (n= 3 independent experiments); ***p* < 0.01, ****p* < 0.001.


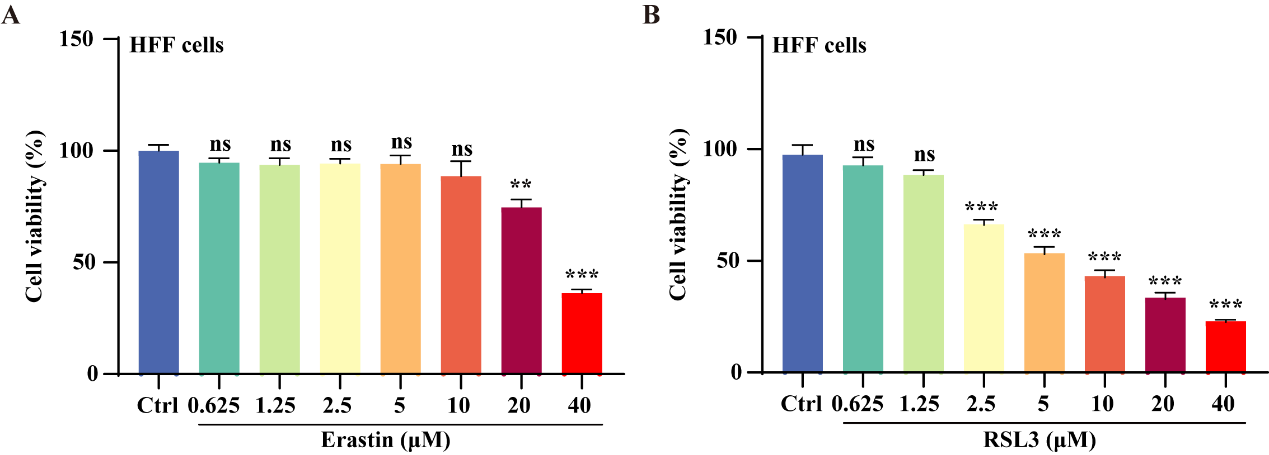


**Figure S2.** **Cell viability of HFF cells treated with Erastin and RSL3. (A-B)** Bar graph showing the viability of HFF cells exposed to different concentrations of Erastin and RSL3. Data are presented as mean ± SD (n= 3 independent experiments); ns *p* > 0.05, ***p* < 0.01, ****p* < 0.001.


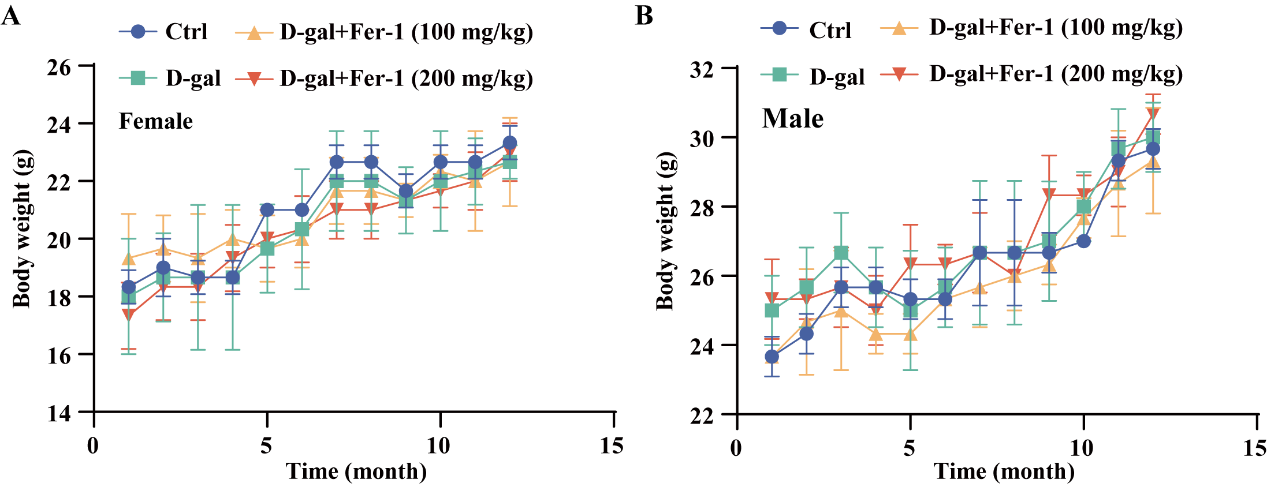


**Figure S3. Assessment of body weight in D-gal-induced C57BL/6J mice treated with Fer-1. (A-B)** Bar graph showing the body weight of female and male C57BL/6J mice subjected to D-gal-induced aging and treated with Fer-1 (100 mg/kg and 200 mg/kg). Data are presented as mean ± SD (n= 6 independent animals); ns *p* > 0.05.

**
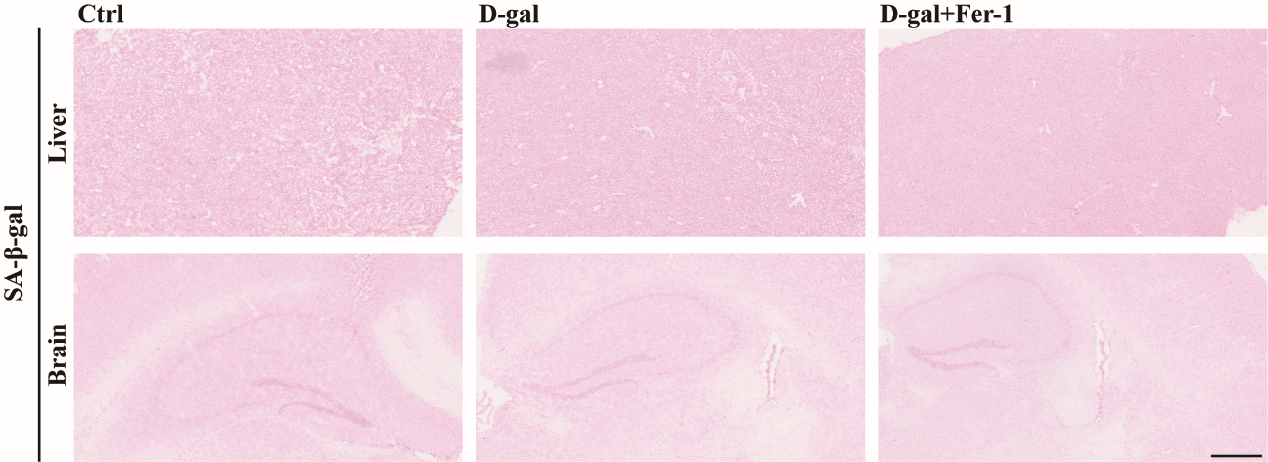
 Figure S4. SA-β-gal-positive staining of the liver and brain tissues in D-gal-induced mice.** SA-β-gal-positive staining was not evident in these tissues. Scale bar: 500 μm.

**
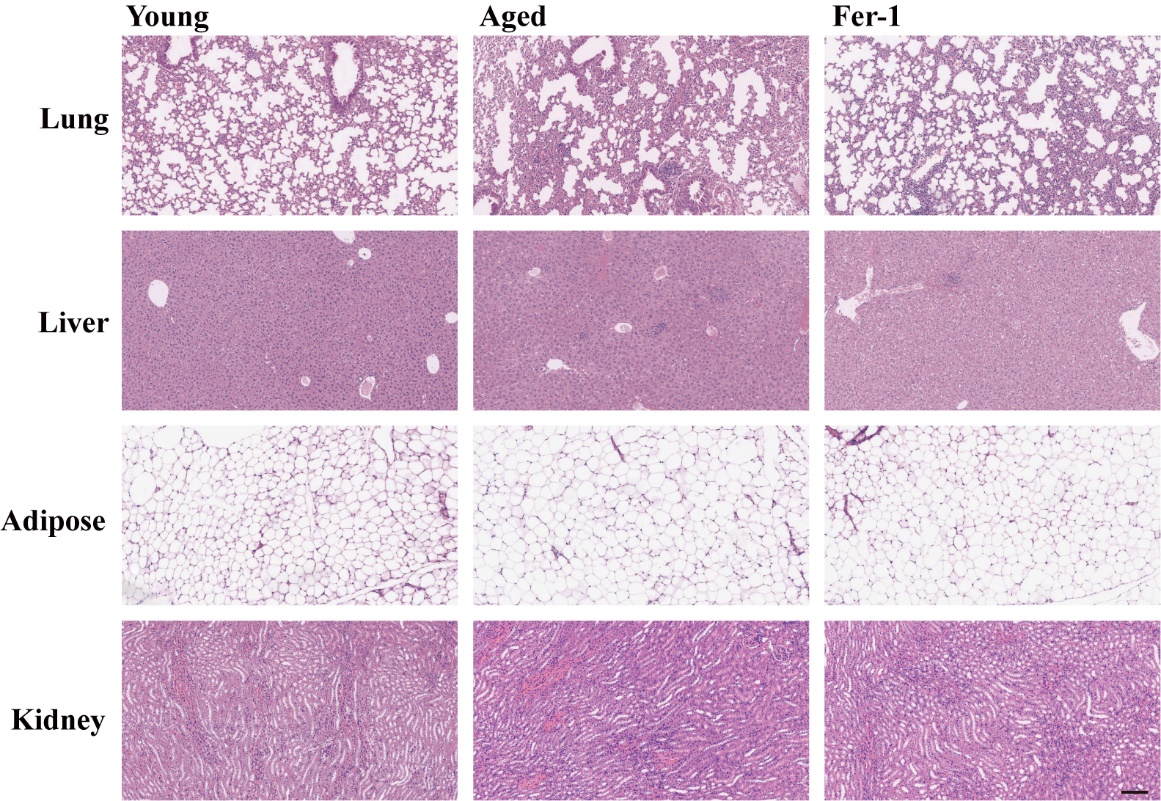
**

**Figure S5. H&E staining of tissues in naturally aged C57BL/6J mice.** H&E staining of lung, liver, adipose, and kidney tissues to observe pathological damage. Scale bars: 200 μm.

**Supplementary Table**

**Table S1. qRT-PCR primer sequences used in this study.**

| Organism | Gene name | Forward Primer（5'→3'） | Reverse Primer（5'→3'） |
| --- | --- | --- | --- |
| *Homo sapiens* | p16 | ATGGAGCCTTCGGCTGACT | GTAACTATTCGGTGCGTTGGG |
|  | p21 | GCCTGTCCACCATCTCCCTAT | TTCAAGCACAGTTATTCTGGACCTT |
|  | IL-6 | ACTCACCTCTTCAGAACGAATTG | CCA TCTTTGGAAGGTTCAGGTTG |
|  | IL-1α | CATTGGCGTTTGAGTCAGCA | GAACTGTCTTCTTCATTTTCACTGT |
|  | IL-1β | CTGTCCTGCGTGTTGAAAGA | TTGGGTAA TTTTTGGGA TCTACA |
|  | Cxcl-3 | GCAGGGAATTCACCTCAAGA | GGTGCTCCCCTTGTTCAGTA |
|  | Mmp-9 | AGACCTGGGCAGATTCCAAAC | CGGCAAGTCTTCCGAGTAGT |
|  | Mmp-12 | CATGAACCGTGAGGATGTTGA | GCATGGGCTAGGATTCCACC |
|  | Cxcl-8 | GAAGTTTTTGAAGAGGGCTGAGA | TGCTTGAAGTTTCACTGGCATC |
|  | GAPDH | CCACTCCTCCACCTTTGAC | ACCCTGTTGCTGTAGCCA |
| *Mus musculus* | p16 | AAAGCGAACTCGAGGAGAGC | AATCGGGGTACGACCGAAAG |
|  | p21 | CCTGGTGATGTCCGACCTG | CCATGAGCGCATCGCAATO |
|  | IL-6 | TGAGAAAAGAGTTGTGCAATGG | GGTACTCCAGAAGACCAGAGG |
|  | IL-10 | GTAGAAGTGATGCCCCAGGC | CACCTTGGTCTTGGAGCTTATT |
|  | IL-1α | AGGGAGTCAACTCATTGGCG | TGGCAGAACTGTAGTCTTCGT |
|  | IL-1β | TGCCACCTTTTGACAGTGATG | AAGGTCCACGGGAAAGACAC |
|  | TNF-α | CCCCAAAGGGATGAGAAGTT | CACTTGGTGGTTTGCTACGA |
|  | NF-kB | CTCTGGCACAGAAGTTGGGT | TCCCGGAGTTCATCTCATAGT |
|  | Cxcl-1 | ACCCAAACCGAAGTCATAGCC | TTGTCAGAAGCCAGCGTTCA |
|  | Cxcl-2 | CCCAGACAGAAGTCATAGCCAC | TGGTTCTTCCGTTGAGGGAC |
|  | Cxcl-3 | ACCCAGACAGAAGTCATAGCCA | CTTCATCATGGTGAGGGGCT |
|  | Mmp-3 | CAGTCCCTCTATGGAACTCCC | AGGGTGCTGACTGCATCAAA |
|  | Mmp-12 | TGCACTCTGCTGAAAGGAGTCT | GTCATTGGAATTCTGTCCTTTCCA |
|  | GAPDH | TGTGATGGGTGTGAACCACGAGAA | GAGCCCTTCCACAATGCCAAAGTT |

**Supplementary Video**

**Video S1: Fer treatment enhances the mobility of *C. elegans* at 5 and 10 days.**
